# Clinical pharmacology of cytotoxic drugs in neonates and infants: Providing evidence-based dosing guidance

**DOI:** 10.1016/j.ejca.2021.11.001

**Published:** 2022-03

**Authors:** A. Laura Nijstad, Shelby Barnett, Arief Lalmohamed, Inez M. Bérénos, Elizabeth Parke, Vickyanne Carruthers, Deborah A. Tweddle, Jordon Kong, C. Michel Zwaan, Alwin D.R. Huitema, Gareth J. Veal

**Affiliations:** a Department of Clinical Pharmacy, University Medical Center Utrecht, Heidelberglaan 100, 3584 CX Utrecht, the Netherlands; b Department of Pharmacology, Princess Máxima Center for Pediatric Oncology, Heidelberglaan 25, 3584 CS Utrecht, the Netherlands; c Newcastle University Centre for Cancer, Newcastle University, NE2 4HH Newcastle Upon Tyne, UK; d Utrecht Institute for Pharmaceutical Sciences, Utrecht University, Universiteitsweg 99, 3584 CG Utrecht, the Netherlands; e Great North Children's Hospital, NE1 4LP Newcastle Upon Tyne, UK; f Division of Cancer Sciences, University of Manchester, Manchester, UK; g Princess Máxima Center for Pediatric Oncology, Heidelberglaan 25, 3584 CS Utrecht, the Netherlands; h Department of Pediatric Oncology, Erasmus MC-Sophia Children's Hospital, Dr. Molewaterplein 40, 3015 GD Rotterdam, the Netherlands; i Department of Pharmacy & Pharmacology, Netherlands Cancer Institute, Plesmanlaan 121, 1066 CX Amsterdam, the Netherlands

**Keywords:** Antineoplastic agents, Child, Clinical protocols, Infant, Medical oncology, Paediatrics, Pharmacokinetics, Pharmacology, Therapeutic drug monitoring

## Abstract

Cancer in neonates and infants is a rare but challenging entity. Treatment is complicated by marked physiological changes during the first year of life, excess rates of toxicity, mortality, and late effects. Dose optimisation of chemotherapeutics may be an important step to improving outcomes. Body size–based dosing is used for most anticancer drugs used in infants. However, dose regimens are generally not evidence based, and dosing strategies are frequently inconsistent between tumour types and treatment protocols. In this review, we collate available pharmacological evidence supporting dosing regimens in infants for a wide range of cytotoxic drugs. A systematic review was conducted, and available data ranked by a level of evidence (1–5) and a grade of recommendation (A–D) provided on a consensus basis, with recommended dosing approaches indicated as appropriate. For 9 of 29 drugs (busulfan, carboplatin, cyclophosphamide, daunorubicin, etoposide, fludarabine, isotretinoin, melphalan and vincristine), grade A was scored, indicating sufficient pharmacological evidence to recommend a dosing algorithm for infants. For busulfan and carboplatin, sufficient data were available to recommend therapeutic drug monitoring in infants. For eight drugs (actinomycin D, blinatumomab, dinutuximab, doxorubicin, mercaptopurine, pegaspargase, thioguanine and topotecan), some pharmacological evidence was available to guide dosing (graded as B). For the remaining drugs, including commonly used agents such as cisplatin, cytarabine, ifosfamide, and methotrexate, pharmacological evidence for dosing in infants was limited or non-existent: grades C and D were scored for 10 and 2 drugs, respectively. The review provides clinically relevant evidence-based dosing guidance for cytotoxic drugs in neonates and infants.

## Highlights

• Treating cancer in neonates and infants is challenging.
• Dose optimisation of cytotoxic drugs is an important step to improving outcomes.
• Clinical pharmacological evidence supporting dosing regimens in infants was collated.
• All available pharmacological evidence was ranked by a level of evidence.
• A grade of recommendation was derived and a recommended dose per agent provided.

## 1 Introduction

Cancer in neonates and infants aged <1 year is a rare entity posing unique challenges. Not only do infants develop different types of cancer; the clinical behaviour, aetiology, biology and prognosis of these cancers differ from older children [1]. Treatment challenges include physiological changes in the first year of life influencing pharmacokinetics, with excess rates of toxicity, mortality and late effects observed in this vulnerable age group [[2], [3], [4], [5]].

Reported incidence of all cancers in the first year of life ranges from 194 to 243 per million, accounting for around 10% of cancer in 0- to 15-year-olds [[6], [7], [8], [9], [10], [11]]. The most common tumours in this age group are neuroblastoma, leukaemia, central nervous system (CNS) tumours, retinoblastoma, and renal tumours (Table 1), with some variation amongst geographic and ethnic groups [6,7,[9], [10], [11], [12]]. Overall survival of infant cancers has improved to around 80% in the last two decades [7,9,10,13]. Survival varies widely between tumour groups, with survival above 80–90% consistently reported in retinoblastoma, neuroblastoma and renal tumours in this age group, but below 50–65% in leukaemia and CNS tumours [7,9,10,[13], [14], [15]]. Historically, efforts to improve survival have relied on intensifying therapy, which is hampered by amplifying the risks of acute toxicity and late effects. Childhood cancer survivors, regardless of age at diagnosis, have increased rates of chronic disease, mental health problems and early death, reduced fertility and lower rates of employment and marriage compared with age-matched controls or siblings [16,17]. Certain late effects, including second neoplasms, need for special education, and impaired growth, occur significantly more frequently amongst children diagnosed at a younger age [[16], [17], [18], [19]].

**Table 1:** Frequency of cancer types in infants aged <1 year reported across registries in France, Israel, Australia, the United States, and the United Kingdom [6,7,[9], [10], [11], [12]].

Cancer type	Incidence per million	% of all diagnoses <1 year
Neuroblastoma	41–58	21–35
Leukaemia	37–40	14–21
CNS tumours	27–34	8–16
Retinoblastoma	18–27	8–13
Renal tumours	17–18	8–11
Germ cell tumours	14	6–9
Liver tumours	5–9	3–5
Total	189–243	

The clinical and biological features of cancer in infancy differ from their older paediatric counterparts. For example, neuroblastoma in older children is typically an aggressive disease, but an infant subtype (stage 4S) exists, which can spontaneously regress, even in the presence of widespread dissemination and is associated with markedly better survival [20,21]. Leukaemia and tumours of the CNS are associated with inferior prognosis and unique treatment challenges in infants. Lymphoid leukaemia occurs more frequently than myeloid leukaemia, although acute myeloid leukaemia (AML), which represents only 16% of all childhood leukaemia, accounts for 35% of infant leukaemia [6,22]. *KMT2A* (previously known as *MLL*) rearrangements occur in up to 80% of acute lymphoblastic leukaemia (ALL) and 50% of AML in infants, compared with 5% and 15% of older children, respectively [22]. Survival in ALL is markedly worse in infants than older children (47% versus 85%), despite the development of novel treatment protocols [22,23]. In contrast, infant event-free survival (EFS) in AML approximates that of older children at around 60%, despite marked biological differences [24].

The treatment of infants and neonates with cancer can be challenging, reflected by a fourfold increase in deaths within 30 days of diagnosis in this age group [[25], [26], [27]]. Increased mortality is in part due to the aggressive biology and advanced presentation of infant tumours but also due to increased toxicity of treatment in this age group. Toxicity is multifactorial, including immaturity of the immune system, organ development and metabolic function. Infectious deaths related to treatment in AML occurred in 13% of children aged <2 years compared with 6% of older children. In the early stages of the CCG1953 ALL study, infectious deaths were seen in 50% of children under 3 months, compared with 18% of 6- to 12-month-olds, leading to dose modifications of daunorubicin [28,29]. Historically, infants with Wilm's tumour or ALL were treated under the same chemotherapy regimens as older children, leading to significantly more multiorgan toxicity in infants [30,31]. This effect was ameliorated by empirical dose reductions, and efforts have since focussed on exploring the pharmacokinetics of chemotherapeutic agents in infants to optimise chemotherapy dosing [32,33].

There are well-established physiological differences between neonates and infants compared with older children who have the potential to significantly impact on drug disposition, and these differences have been comprehensively covered in previous publications [[34], [35], [36]]. These differences include age-dependent changes in gastrointestinal tract structure and function, which may impact on drug absorption, developmental changes in percentages of total body water and body fat alongside differences in plasma protein binding affecting drug distribution, changes in metabolic capacity related to the ontogeny of enzymes involved in drug metabolism and physiological developmental changes in kidney function impacting drug elimination. Clearly, these differences need to be taken into account when considering the dosing of chemotherapeutics in the neonate and infant patient population.

Infants with cancer represent a unique group with different biological drivers to cancer in older children. Many of these cancers are aggressive and require unique treatment approaches. At the same time, these children are uniquely vulnerable to the effects of treatment. Developing approaches to optimise exposure to chemotherapeutic drugs may represent an important step to improving outcomes in this challenging group. The chemotherapeutic agents used in the commonest infant cancers are listed in Table 2.

**Table 2:** Common infant cancers and current first-line chemotherapy agents.

Cancer type	Chemotherapy drugs used
Acute lymphoblastic leukaemia	Cyclophosphamide, cytarabine, daunorubicin, dexamethasone, etoposide, mercaptopurine, methotrexate, PEG-asparaginase, prednisone, thioguanine, vincristine, triple intrathecals (methotrexate, cytarabine, prednisone)
Acute myeloid leukaemia	Cytarabine, fludarabine, gemtuzumab, idarubicin, mitoxantrone
Neuroblastoma	Busulfan, carboplatin, cisplatin, cyclophosphamide, dinutuximab, doxorubicin, etoposide, isotretinoin, melphalan, topotecan, vincristine
Retinoblastoma	Carboplatin, etoposide, vincristine, intrathecal cytarabine
Wilms tumour	Actinomycin D, carboplatin, cyclophosphamide, doxorubicin, etoposide, vincristine

## 2 Current approaches to the dosing and the application of pharmacological data

For the vast majority of anticancer drugs used in neonates and infants, dosing regimens based on body weight are used in the clinic. This is partly a practical consideration as body surface area (BSA) is more challenging to predict accurately in this population compared with body weight and partly because of the tendency to overdose neonates and infants, since the developmental changes in pharmacokinetic parameters do not change proportionally with BSA. However, the body weight–based doses incorporate a discrepancy in dose compared with the equivalent BSA-based dose administered to children aged >1 year, or >10 or 12 kg, depending on the drug and clinical protocol on which the child is being treated. Dose adjustments for infants are frequently used inconsistently between tumour types and treatment protocols, with additional dose reductions of 33–50% commonly recommended for children aged <6 months or <5 kg, for example. This subject has been previously discussed in a number of well-written review papers, highlighting the lack of clinical pharmacological data supporting many current dosing regimens and the marked dose increases implemented for many anticancer drugs when infants cross a dosing threshold boundary of 12 kg or 1 year of age [4,33,37]. As an example of the current state of play for the widely used anticancer drug vincristine, Table 3 provides examples of dosing regimens and recommended dose reductions for infants and neonates across a range of tumour types. As can be seen, clear inconsistencies exist between tumour type as to the most appropriate dosing regimens and adjustments for infant cancer patients of varying ages compared with the standard BSA-based dosing in older children. The one thing that is likely to be consistent across treatment protocols is that none of the dose reductions stipulated for infant patients is based on any kind of meaningful pharmacological rationale. To avoid the current situation whereby marked dose increments are introduced when infants cross defined weight or age boundaries, the COG Chemotherapy Standardization Task Force has recently recommended the use of dosing tables for infants to gradually transition from body weight to BSA-based dosing [32]. While potentially useful, these guidelines are, as acknowledged by the authors, a temporary solution designed to improve the current infant dosing situation in the absence of more rational-based adaptive dosing approaches.

**Table 3:** Vincristine dosing regimens and dose adjustments across a range of tumour types.

Tumour type	Dose	Route	Dose adjustment	Absolute dose for a child of:		
				2 months, 5.5 kg, 0.30 m^2^	6 months, 8 kg, 0.39 m^2^	12 months, 10 kg, 0.46 m^2^
Ependymoma (postoperative intensive chemotherapy) and infant ependymoma	1.5 mg/m^2^	IV infusion (1 h)	Children > 12 months: use full BSA-based dose (1.5 mg/m^2^) For children 6–11 months and over: use 75% of BSA-based dose (1.125 mg/m^2^) For children 6 months and under: use 50% BSA-based dose (0.75 mg/m^2^)	0.22 mg	0.44 mg	0.69 mg
Low-grade glioma (induction therapy)	1.5 mg/m^2^	IV bolus	For children < 10 kg: 0.05 mg/kg/day For children <6 months: further dose reduction of 33%	0.18 mg	0.40 mg	0.69 mg
Low-grade glioma (consolidation therapy)	1.5 mg/m^2^	IV bolus	For children <10 kg: 0.05 mg/kg/day For children < 6 months: further dose reduction of 33%	0.18 mg	0.40 mg	0.69 mg
Low-risk medulloblastoma	1.5 mg/m^2^	IV bolus	For children 12 months and over: use full BSA-based dose (max 1.5 mg/m^2^) For children 6–11 months and over: use 80% of BSA-based dose (1.2 mg/m^2^) For children 6 months and under: use 66% BSA-based dose (0.99 mg/m^2^)	0.30 mg	0.47 mg	0.69 mg
Non-metastatic rhabdomyosarcoma	1.5 mg/m^2^	IV bolus	For children <12 months or <10 kg: 0.05 mg/kg/day	0.28 mg	0.40 mg	0.69 mg
Relapsed/refractory rhabdomyosarcoma	1.5 mg/m^2^	IV bolus	For children <10 kg: 0.05 mg/kg/day	0.28 mg	0.40 mg	0.69 mg
High-risk neuroblastoma	1.5 mg/m^2^	IV bolus	For children < 12 kg, use 0.05 mg/kg For infants < 5 kg, a further 33% reduction is recommended	0.28 mg	0.40 mg	0.50 mg
High-risk neuroblastoma (second-line schema)	2 mg/m^2^	Continuous IV infusion (48h)	For children < 12 kg: use 0.033 mg/kg/day For infants < 5 kg: a further 33% reduction is recommended	0.36 mg	0.52 mg	0.66 mg
Relapsed/progressive high-risk neuroblastoma	1 mg/m^2^	Continuous IV infusion (48h)	For children <12 kg: use 0.033 mg/kg	0.18 mg	0.26 mg	0.33 mg
Low/intermediate-risk neuroblastoma	1.5 mg/m^2^	IV bolus	For children < 10 kg: use 0.05 mg/kg For infants below 5 kg: reduce by a further 33%	0.28 mg	0.40 mg	0.69 mg

BSA, body surface area; IV, intravenous.

There are good reasons why dose reductions may be needed in the infant cancer patient, either related to a reduced drug clearance associated with the early development of kidney and liver function in the first weeks and months of life or due to an increased susceptibility to adverse drug effects in the developing child. However, with the critical importance of getting the balance right between efficacy and toxicity in this patient population, it would be prudent to consider pharmacological evidence to either support or refute current dosing regimens where this is available. A good example of how data generated from clinical pharmacological studies can be used to improve dosing practices is provided by the use of 13-cis-retinoic acid in a high-risk neuroblastoma setting. A study designed to investigate the feasibility of using therapeutic drug monitoring (TDM) approaches to 13-cis-retinoic acid dosing showed marked variability in drug exposures between patients and highlighted that children <12 kg who were receiving a body weight–based drug dose were achieving consistently low and potentially subtherapeutic drug levels [38]. The findings from this study led to the removal of body weight–based dosing regimens for the younger patients, with all patients across Europe now receiving the standard BSA-based dose, with no reported issues in terms of tolerability. The study also had the added benefit of stimulating research that led to the recent development of an infant friendly liquid formulation of the drug [39].

Although more prospective studies are needed in this area, incorporating relevant pharmacokinetic and pharmacodynamic end-points to generate data that can inform the selection of dosing regimens in neonates and infants, it is also important to scrutinise the currently available literature to investigate what current evidence is available. This information should be looked at alongside patient characteristics that may be used to determine more rational dosing regimens in neonates and infants. Such characteristics may include gestational or postnatal age, ontogeny information relating to metabolic and elimination processes, and renal function measurements and body weight.

## 3 Pharmacokinetics of selected chemotherapeutics in neonates and infants

**Fig. 1:**
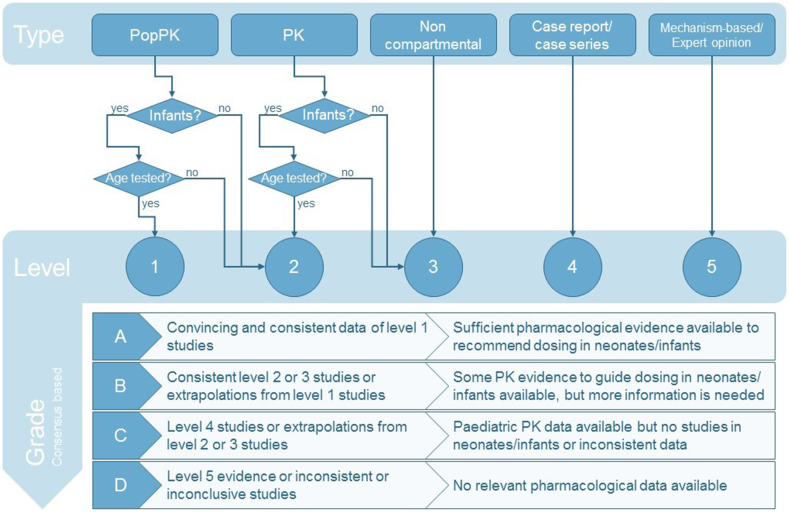
Graphical summary of the methods used for labelling articles with a specific level and grading of the chemotherapeutic agents.

**Fig. 2:**
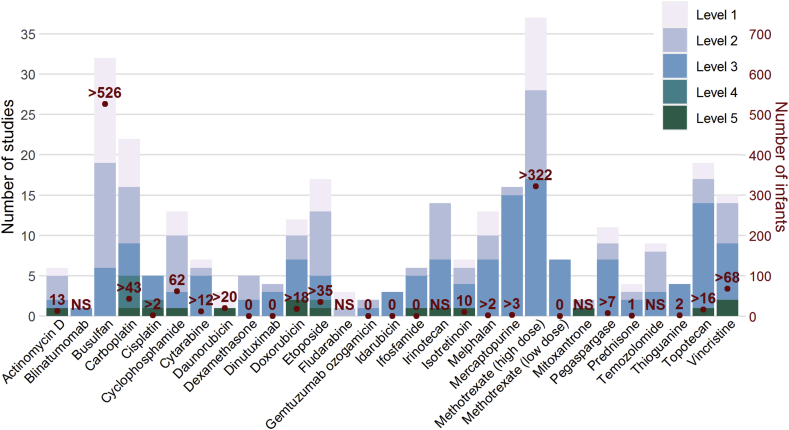
Bar plot displaying the number of studies for each chemotherapeutic agent per evidence level (primary y-axis), as well as the number of infants included in total in all published studies (secondary y-axis).

Many chemotherapeutic agents are used in infants, despite pharmacological evidence for the dosing regimens used being scarce or even non-existent for the majority of anticancer drugs. For the present study, we investigated and collated the available pharmacological evidence supporting dosing regimens in infants and neonates for a wide range of clinically relevant cytotoxic drugs. A graphical summary of the workflow is shown in Fig. 1, with levels of evidence and grades of recommendation inspired by the Oxford Centre for Evidence-Based Medicine system, as outlined in the detailed methods provided in Supplementary file 1. All available pharmacological evidence was ranked based on the level of evidence (1–5) (Supplementary file 2, Table S5). Subsequently, a grade of recommendation (A–D) and a recommended dose per chemotherapeutic agent was derived by consensus opinion. For grade C or D agents, no dose advice is given because the pharmacological evidence was insufficient to come to a recommendation. Fig. 2 gives an overview of the available pharmacological evidence per level for each chemotherapeutic agent of interest, alongside the total number of infants studied in the available papers.

Our recommendations for dosing regimens for chemotherapeutic agents in neonates and infants are summarised in Table 4 and discussed below for each of the grades of classification. A comprehensive list of publications and reference details for each drug are provided in Table S5 (Supplementary file 2), with key references included within the sections below. To provide examples of how the results from clinical pharmacology studies can positively impact on clinical practice, we describe in detail two drugs (carboplatin and busulfan) classified as grade A, for which pharmacokinetic data are well understood and are used to provide ‘gold standard’ treatment. Both drugs meet the criteria for TDM (e.g. narrow therapeutic index, a clear relation between exposure and clinical outcome, substantial interpatient variability and small intrapatient variability), and evidence shows that TDM practices can be successfully used to optimise the treatment for neonate and infant patients.

**Table 4:** Results of the studied chemotherapeutic agents with the recommendations for dosing regimens in neonates and infants.

Chemotherapeutic agent	PK findings/remarks	Recommended dosing regimen and dose adjustments for infants		Grade of recommendation
Actinomycin D	Some PK studies in children have been published, one including infants. The results on the effect of age on the PK are not consistent.	Full (mg/m^2^) dose		B
Blinatumomab	Two PK studies in children have been published. No effect of age on the PK of blinatumomab has been found. However, the PK behaviour of antibodies in infants is known.	Full (mg/m^2^) dose		B
Busulfan	Busulfan has been thoroughly studied in infants. It demonstrates a U-shaped relationship between age and clearance. TDM-guided dosing is associated with higher event-free survival rates due to fewer graft failures or relapses and lower toxicity.	Dose for day 1 (in case of a target AUC of 90 mg/l∗h), after which the dose is adjusted based on TDM:		A
		**BW (kg)**	**Dose 1dd (mg/kg)**	
		3	3.8	
		5	4.7	
		7	5.1	
		8–13	5.2	
		15–16	5.1	
Carboplatin	The PK of carboplatin in children has been studied thoroughly. The results on the effect of age on the PK are not consistent in all studies. TDM-guided dosing has been successfully implemented in the United Kingdom.	Use TDM approach to achieve target AUC. If not available dose based on mg/kg or GFR.		A
Cisplatin	Some PK studies in children have been published, including one case report on an infant. However, the level of evidence for a specific dose regimen is low. CL might be lower in younger patients.	*No advice*		C
Cyclophosphamide	PK studies in children (including infants) have been published. A higher CL had been found in younger children, resulting in a higher exposure to metabolites.	Use mg/m^2^ dose, reduce by 20% in young infants (<6 months)		A
Cytarabine	Some PK studies in children have been published; however, the number of infants was limited, and the effect of age was not studied in most of the studies.	*No advice*		C
Daunorubicin	Two PK studies including infants have been published. No effect of age on the PK of daunorubicin observed.	Full (mg/m^2^) dose		A
Dexamethasone	Some PK studies in children have been published; however, no infants were included. CL might be higher in younger patients.	*No advice*		C
Dinutuximab	Some PK studies in children have been published; however, no infants were included, and the effect of age was not studied in most of the studies. However, the pharmacokinetic behaviour of antibodies in infants is known. CL might be higher in younger patients.	Full (mg/m^2^) dose		B
Doxorubicin	The PK of doxorubicin has been investigated in infants. A lower CL of doxorubicin has been found in younger patients; however, the number of infants included are low.	Adapt the dose based on age and BSA and duration of infusion, according to equations in Siebel *et al.* (2020) [55].		B
Etoposide	PK studies including infants have been published. No effect of age on the PK of etoposide has been found.	Full (mg/m^2^) dose		A
Fludarabine	Some PK studies in children have been published; however, the number of infants was limited. No effect of age on the PK of fludarabine has been found.	Full (mg/m^2^) dose. Consider dose adaptation based on eGFR in case of renal impairment.		A
Gemtuzumab ozogamicin	Some PK studies in children have been published; however, no infants were included. No effect of age on the PK of gemtuzumab ozogamicin observed.	*No advice*		C
Idarubicin	Some PK studies in children have been published; however, no infants were included, and the effect of age was only studied once. No effect on the PK of idarubicin has been found.	*No advice*		C
Ifosfamide	Some PK studies in children have been published; however, no infants were included, and the effect of age on the PK parameters was only studied once. No effect on the PK of ifosfamide has been found.	*No advice*		C
Irinotecan	PK studies in children (including infants) have been published. The effect of age was not studied in most of the studies. CL of the metabolite might be higher in younger children.	*No advice*		C
Isotretinoin	Some PK studies including infants have been published. No effect of age on the PK of isotretinoin has been found.	Full (mg/m^2^) dose		A
Melphalan	PK studies in children (including infants) have been published. No effect of age on the PK of melphalan has been found.	Full (mg/m^2^) dose		A
Mercaptopurine	PK studies in children have been published; however, the number of infants was limited. No effect of age on the PK of mercaptopurine has been found.	Full (mg/m^2^) dose, adjust based on WBC.		B
Methotrexate (high dose)	The PK of high-dose methotrexate has been investigated in infants. However, these studies show conflicting results.	*No advice*		D
Methotrexate (low dose)	Some PK studies in children have been published; however, no infants were included, and the effect of age was not studied in most of the studies. No effect of age on the PK of methotrexate low dose has been found.	*No advice*		C
Mitoxantrone	One PK study in children has been published (unknown number of infants). The effect of age on the PK of mitoxantrone has not been studied.	*No advice*		D
Pegaspargase	PK studies in children have been published; however, the number of infants was limited, and the effect of age was not studied in most of the studies. No effect of age on the PK of pegaspargase has been found.	Full (mg/m^2^) dose, adjust based on TDM.		B
Prednisone	Some PK studies in children have been published; however, no infants were included. No effect of age on the PK of prednisolone has been found.	*No advice*		C
Temozolomide	Some PK studies in children have been published; however, the number of infants was limited, and the effect of age was not studied in most of the studies.	*No advice*		C
Thioguanine	Some PK studies in children have been published; however, the number of infants was limited, and the effect of age was not studied in most of the studies. No effect of age on the PK of thioguanine has been found.	Full (mg/m^2^) dose, adjust based on WBC.		B
Topotecan	PK studies in children (including infants) have been published; however, the effect of age was not studied in most of the studies. The studies on the effect of age on the PK of topotecan show conflicting results.	Full (mg/m^2^) dose		B
Vincristine	PK studies in children have been published; however, the number of infants was limited. Most studies did not find an effect of age on the PK.	Full mg/m^2^ or mg/kg dose (≥0.05 mg/kg). For neonates (0–4 weeks of age), use mg/kg dose (≥0.05 mg/kg).		A

AUC, area under the curve; BSA, body surface area; BW, body weight; CL, clearance; GFR, glomerular filtration rate; NS, not specified; PK, pharmacokinetics; SCT, stem cell transplantation; TDM, therapeutic drug monitoring; WBC, white blood cell count.

### 3.1 Grade A

For nine agents (busulfan, carboplatin, cyclophosphamide, daunorubicin, etoposide, fludarabine, isotretinoin, melphalan and vincristine), a grade A recommendation was given, for which sufficient pharmacological evidence is available to recommend dosing in infants. Details on busulfan and carboplatin are discussed separately below.

For daunorubicin, etoposide, isotretinoin and melphalan, sufficient and consistent level 1 pharmacokinetic studies including infants have been published (Supplementary file 2, Table S5). For all these drugs, no effect of age on pharmacokinetics has been observed, and a full (mg/m^2^) dose is recommended.

Several studies on the pharmacokinetics of cyclophosphamide in children have been published, including a total of 62 infant patients. No structural effect of age was found on pharmacokinetic parameters, but in two level 1 population pharmacokinetic analyses including a total of 54 infants, a higher clearance was observed in younger children, resulting in a greater exposure to active metabolites [40,41]. Therefore, a recommendation to use the mg/m^2^ dose and reduce the dose by 20% in younger infants (<6 months) is supported, as proposed by Campagne *et al.* [40].

Although relatively few studies reporting on fludarabine pharmacokinetics in children have been published, and the number of infants included in these studies was unspecified, the quality of the analyses was high (two level 1 studies), and all studied the effect of age. No effect of age on fludarabine pharmacokinetics was found, but estimated glomerular filtration rate (eGFR) was included as a significant covariate for clearance in all studies [[42], [43], [44]]. A recommendation to administer the full (mg/m^2^) dose is supported, with dose adaptation based on eGFR in cases of renal impairment.

Several vincristine pharmacokinetic studies in children have been conducted over the past 25 years, with a recently published level 1 population study focussing on drug disposition in neonates and infants, including 21 patients aged <1 year [45]. No significant difference in BSA-normalised clearance between infants and older children was found in this study; however, there was a trend towards lower clearance in neonates (0–4 weeks) compared with infants (1–12 months). Doses of <0.05 mg/kg resulted in significantly lower area under the plasma concentration-time curve (AUC) values than observed in neonates and infants receiving doses of ≥0.05 mg/kg and older children receiving a dose of 1.5 mg/m^2^. No significant differences in vincristine exposures between younger patients receiving vincristine doses of ≥0.05 mg/kg and older children (1.5 mg/m^2^) were observed. These findings are supported by previously published level 2 and 3 studies, which did not find an effect of age on vincristine pharmacokinetics. These recent data support a recommendation of either full (mg/m^2^) dosing, or body weight-based dosing at doses of ≥0.05 mg/kg, with the latter approach potentially more appropriate for neonate patients (0–4 weeks of age).

### 3.2 Grade B

For a total of eight drugs (actinomycin D, blinatumomab, dinutuximab, doxorubicin, mercaptopurine, pegaspargase, thioguanine and topotecan), the available pharmacological evidence to guide dosing in infants was classified as grade B.

For actinomycin D, some pharmacokinetic data in children are available, with 13 infants included across two studies [46,47]. However, conclusions drawn on the effect of age on the pharmacokinetics of actinomycin D are inconsistent. This could be because of different analytical methods used or the limited number of infants included. More information is needed to provide an evidence-based actinomycin D dose recommendation. Until such information is available, it is recommended that the full (mg/m^2^) dose is administered. This is based on the findings of a non-compartmental analysis, where Skolnik *et al.* found that clearance, corrected for BSA, was not related to age [47].

Some pharmacokinetic studies have been published in children focussing on the monoclonal antibody drugs blinatumomab and dinutuximab. For blinatumomab, a limited number of infants were included, and no effect of age on pharmacokinetics was found [48,49]. For dinutuximab, no infants were studied, and the effect of age was not investigated in the majority of studies, although there was a suggestion that dinutuximab clearance may be higher in younger patients [50]. Although the pharmacokinetic behaviour of antibodies in infants, in general, is reasonably well studied, specific information on the pharmacokinetics of blinatumomab and dinutuximab is limited. In accordance with current practice, a full (mg/m^2^) dose for blinatumomab and dinutuximab is recommended in infants and neonate patients.

The pharmacokinetics of doxorubicin have been investigated in infants, with a lower clearance of doxorubicin observed in younger patients [[51], [52], [53], [54]]. However, a limited number of infant patients were included in these studies. In addition, pharmacokinetic simulations using a published population pharmacokinetic model were performed by Siebel *et al.* [55]. Equations for individualisation of the doxorubicin dose based on age and BSA were published, accompanied by the advice to reduce the peak concentrations in very young children by prolonging drug infusion. As this analysis is based on a population pharmacokinetic model including only four infants, it is recommended that these findings are confirmed in a larger infant patient cohort.

Mercaptopurine and thioguanine pharmacokinetic studies in children have been published, although the number of infant patients included is limited to a handful of studies [51,56,57]. No effect of age on the pharmacokinetics of mercaptopurine has been found, with the effect of age on the pharmacokinetics of thioguanine not investigated in most of the studies. More information on the pharmacokinetics of both of these drugs in infants and neonates is needed to further elucidate the effect of age on drug disposition. Based on current practice, a full (mg/m^2^) dose is recommended, with dose adjustments based on white blood cell count.

For pegaspargase, although several pharmacokinetic studies in children have been published, number of infant patients included is limited, and the effect of age was not investigated in most cases. Although preliminary data would suggest no effect of age on pegaspargase pharmacokinetics [[58], [59], [60], [61]], more studies including infant patients are needed to provide evidence-based dosing advice. In the meantime, full (mg/m^2^) doses are recommended, with dose adjustments based on TDM approaches.

Topotecan represents an anticancer drug well studied in children. However, the effect of age was not investigated in the majority of published studies. Two level 1 studies including infants, both describing topotecan disposition using population pharmacokinetic models, show conflicting results. Schaiquevich *et al.* found a correlation between age and BSA-normalised clearance and volume of distribution of the central compartment, whereas Roberts *et al.* did not observe any effect of age after normalising for BSA [62,63]. Previous level 3 studies that studied the effect of age, but did not include infants, did not find a correlation between age and pharmacokinetic parameters. There is currently insufficient evidence to recommend changes to currently accepted dosing regimens, which may be based on BSA or BW for different tumour types.

### 3.3 Grade C

Drugs classified as grade C represent those for which paediatric data are available, but where no pharmacological studies have been conducted in infants or where the published data are inconsistent. No dose advice can be provided for these ten agents (cisplatin, cytarabine, dexamethasone, gemtuzumab ozogamicin, idarubicin, ifosfamide, irinotecan, low-dose methotrexate, prednisone and temozolomide) based on a pharmacological rationale.

For cisplatin, some level 3 pharmacokinetic studies including infant patients have been published, and one case report in a neonate [[64], [65], [66]]. The level of evidence for a specific dose regimen is low. Clearance may be lower in younger children, but this needs to be verified in a cohort including infant patients.

Some pharmacokinetic studies of cytarabine in children have been published; however, the number of infants included are limited, and the effect of age is not studied in the majority of cases. The studies that did look into the effect of age reported conflicting results. Although a level 1 study included age as covariate on all pharmacokinetic parameters, a level 2 study failed to observe a change in drug clearance in infants compared with older children [51,67]. Population pharmacokinetic analyses looking into the effect of age on the pharmacokinetics of cytarabine (and metabolites) are needed.

For dexamethasone and prednisone, some pharmacokinetic studies in children have been published in an oncology setting; however, no infant patients were included. Although it has been suggested that dexamethasone clearance may be higher in younger patients [68,69], this finding needs to be verified using a population pharmacokinetic model approach in a study including infants. No correlation between age and BSA-normalised prednisone clearance was reported in a level 1 study incorporating a population pharmacokinetic modelling approach and including a single infant patient [70]. However, plasma protein binding of prednisone to corticosteroid-binding globulin was associated with patient age. These findings need to be examined in a larger cohort of infant patients to provide evidence-based dosing advice.

For gemtuzumab ozogamicin, idarubicin and ifosfamide, only small numbers of pharmacokinetic studies have been published in children, with no infant patients included. No effect of age on the pharmacokinetics of gemtuzumab ozogamicin was observed in two separate studies [71,72], and a single level 3 study on the pharmacokinetics of idarubicin similarly observed no effect of age [73]. The only published ifosfamide population pharmacokinetic model failed to look into the effect of age, and the published level 3 studies did not find an effect of age on ifosfamide pharmacokinetics [74,75]. These findings require verification in population pharmacokinetic studies including infant patients.

Irinotecan pharmacokinetic studies in children, including infant patients, have been published, but frequently not investigating the effect of age. Clearance of the metabolite may be higher in younger children [76], but this finding needs to be verified through studies incorporating population pharmacokinetic model approaches across the paediatric age spectrum.

For low-dose methotrexate, several studies investigating pharmacokinetics in children have been published, but no infants were included, and the effect of age was not studied in the majority of cases. Level 3 non-compartmental studies that did look into the effect of age on pharmacokinetics did not find an effect [[77], [78], [79], [80]]; however, population pharmacokinetic analyses for low-dose methotrexate are needed. Again, for temozolomide, some pharmacokinetic studies have been published in children, but numbers of infant patients were limited or not specified, and the effect of age was not investigated in most cases. The results of one level 1 population pharmacokinetic study, suggesting that age has an effect on BSA-normalised clearance and volume of distribution, did not match the results of two level 3 non-compartmental analyses, which indicated no effect of age on BSA-normalised drug clearance [[81], [82], [83]]. Population pharmacokinetic analyses looking into the effect of age on the pharmacokinetics of temozolomide are needed.

### 3.4 Grade D

The remaining two agents, high-dose methotrexate and mitoxantrone, were classified as grade D, with no relevant pharmacological data currently available or conflicting results published.

Numerous studies on the pharmacokinetics of high-dose methotrexate have been published, and many of these studies included infants. However, the results of these studies are conflicting. Several level 1 and level 2 studies describe no effect of age on pharmacokinetics after including other covariates, such as body weight (using allometric scaling), SLCO1B1 polymorphism, serum creatinine and/or treatment with dexamethasone [[84], [85], [86], [87], [88], [89], [90]]. Nevertheless, some level 1 studies did report an effect of age on high-dose methotrexate clearance or volume of distribution of the central compartment, even after normalising for body size [[91], [92], [93], [94]]. In addition, one-, two- and three-compartment models have been published, suggesting a lack of consensus between studies [84,85,[94], [95], [96], [97], [98], [99],[86], [87], [88], [89], [90], [91], [92], [93]]. These conflicting results could be related to variations in the method of drug analysis or differences in sampling times between the studies, with many models based on data obtained for routine patient care, for example, blood samples taken every 24 h to monitor the plasma concentrations for rescue therapy. The development of population pharmacokinetic models incorporating more intensive sampling times in children and infants is recommended.

For mitoxantrone, only one pharmacokinetic study has been published in children [100], and the effect of age on the mitoxantrone pharmacokinetics has not been studied. No relevant data are available to give an evidence-based dosing regimen.

### 3.5 Carboplatin

Carboplatin is a platinum-based chemotherapeutic agent used to treat a variety of tumour types. It represents a cytotoxic drug for which TDM is well established, with defined target exposures for different tumour types and chemotherapy regimens [101,102]. There is a clear understanding from both adult and paediatric studies of the correlation between exposure of free carboplatin and toxicity/response [103,104], which can be used to obtain optimal exposure and limit the occurrence/severity of side-effects in patients.

Carboplatin elimination is highly dependent on renal function. The glomerular filtration rate (GFR) is used to calculate the dose administered to patients, as proposed by Calvert and Newell [102,105]. These dosing equations are described in greater detail in a recent review by Barnett *et al.* [106]. However, carboplatin dosing based on renal function poses a substantial challenge in neonates and infants because a reliable estimate of GFR is often unavailable. In addition, there is no standardised method of GFR determination across treatment centres, which can lead to marked variations in dose calculation [107]. Therefore, alternative strategies, such as dosing based on BSA, have been developed, where an AUC of 1.325 mg/mL∗min is typically achieved per 100 mg/m^2^ dosed [102]. This mg/m^2^ dosing approach is common for carboplatin paediatric dosing regimens within the United Kingdom [106].

Several studies have highlighted that this strategy might, however, not be appropriate for neonates and infants. Allen *et al.* demonstrated that in children with retinoblastoma, doses of carboplatin were generally higher in those dosed according to mg/m^2^ relative to GFR [108]. Moreover, children who were dosed according to BSA were three times more likely to require a platelet transfusion. It was noted that there was a greater difference in the doses calculated using GFR versus mg/m^2^ for the younger children recruited onto the study. This reflects the marked changes in GFR that occur within the first few months of life and the important role renal function plays in carboplatin elimination.

Barnett *et al.* recently compiled a summary of carboplatin dosing regimens used for various tumour types within the United Kingdom, including the dose reductions that are applied for the treatment for infants/neonates [106]. To illustrate, for low/intermediate-risk neuroblastoma, standard carboplatin dosing is 200 mg/m^2^ to achieve a target AUC of 2.6 mg/mL∗min per day. However, for patients less than 10 kg, a dose of 6.6 mg/kg is administered, and for infants less than 5 kg, this dose is reduced further to 4.4 mg/kg. Therefore, patients <10 kg on 6.6 mg/kg dosing receive 41–67% of the carboplatin dose that would have been administered using 200 mg/m^2^ dosing. These mg/kg adjustments are required for neonates/infants as obtaining accurate estimates of BSA, and GFR can be challenging, and mg/m^2^ dosing has been shown to substantially overestimate the dose required during the first few months of life [108]. In this respect, Qaddoumi *et al.* also showed that younger patients (aged <6 months) had a higher incidence of ototoxicity relative to older patients, most likely as a result of BSA-based carboplatin dosing [109]. Therefore, an emphasis has been placed on the avoidance of this approach in younger patients (<10 kg), in favour of mg/kg dosing [110].

Although dosing in mg/kg can lead to more appropriate doses for carboplatin in neonates and infants, it is not without its limitations. Veal *et al.* showed marked differences in carboplatin clearance between neonates of a similar age and weight over several cycles of treatment [111]. For these patients treated over three cycles, carboplatin clearance increased to a higher magnitude than body weight. Therefore, markedly higher doses than those based solely on changes in body weight were frequently required to achieve carboplatin target AUC, demonstrating the importance of TDM for neonates to attain optimal carboplatin exposures.

Given the limitations, in the United Kingdom, carboplatin TDM is now routinely used for infant neuroblastoma and retinoblastoma patients, as recommended by national treatment guidelines. Details of how this process is carried out have recently been summarised [106]. Target carboplatin exposure depends on tumour type and/or risk group, with doses adjusted accordingly over multiple days of treatment to achieve these targets. For standard carboplatin chemotherapy in neonates and infants, the target AUC typically ranges from 5.2 to 7.8 mg/mL∗min over 3 days. In addition to variations in dose due to tumour type, dose reductions are often applied to children <6 months of age, <12 months of age or less than 10 kg.

### 3.6 Busulfan

Busulfan is an alkylating agent used in conditioning regimens to prepare for both autologous and allogeneic haematopoetic stem cell transplantation. Its pharmacokinetics, pharmacodynamics and pharmacogenetics in this paediatric population have been extensively reviewed by ten Brink *et al.* [112]. Several studies published over many years have shown that busulfan dosing can be optimised by performing TDM [112]. Busulfan, combined with TDM-guided dosing, is associated with higher event-free survival rates due to fewer graft failures or relapses and lower toxicity. The most appropriate target busulfan AUC has been studied in several papers [[113], [114], [115], [116], [117]] and has been optimised over many years, leading to consensus on a target AUC of 78–101 mg∗h/L when combined with fludarabine [116].

Busulfan is one of the relatively few agents that has been thoroughly studied in infants (Supplementary Table S5) and demonstrates a U-shaped relationship between age and clearance. In one of the first busulfan pharmacokinetic papers in infants, Dalle *et al.* describe that exposure in infants can be higher than in older children after similar dosing regimens [118]. In contrast, several papers, published some years later, showed a higher clearance (corrected for body size) of busulfan in children aged <4 years [[119], [120], [121]]. More recent population pharmacokinetic models indicate that clearance (corrected for body size) of busulfan increases after birth until the age of 2–12 years (depending on the pharmacokinetic model) and then begins to decline to adult levels [[122], [123], [124], [125], [126]]. Besides growth, one of the explanations for this increase in the first years of age is maturation of glutathione by glutathione S-transferase (GST) enzymes. Busulfan is extensively metabolised by GST enzymes, predominantly GSTA1. The GST enzymes involved can undergo significant changes in activity and/or expression, increasing gradually over the first 2 years of life [127,128]. In addition, several investigators studied the effect of GSTA1 genetic variations on the pharmacokinetics of busulfan, and GSTA1 genetic variations were incorporated into population pharmacokinetic models in children and adults [[129], [130], [131], [132], [133], [134], [135]].

These insights into the pharmacokinetics of busulfan in children and, in particular, infants have led to the development of several age-based dosing strategies for initial busulfan dosing regimens (in mg/kg), whereafter the dose is adjusted based on TDM [122,[136], [137], [138], [139], [140]]. Current dosing recommendations from the European Medicines Agency and the Food and Drug Administration for the use of busulfan in children are based on the nomogram of Nguyen *et al.*, which is based on a pharmacokinetic model that takes only body weight into account, although more recent population pharmacokinetic models suggest that maturation should also be considered [136,141].

Studies pointed out that there is no difference in pharmacokinetics between dosing once daily or multiple times per day [114,124,142]. The exposure to busulfan can be adequately calculated based on 2–4 plasma levels, which is minimally invasive and does not exceed the limits of blood withdrawal in infants [112,143].

## 4 Future directions

In the current review, we have collated data from clinical pharmacological studies incorporating pharmacokinetic data of cytotoxic agents in neonates and infants, with many of these studies involving the recruitment of only small numbers of individuals in the very young age category. Although we have attempted to use this information to provide guidance for future dosing of infants with the selected drugs, there is still clearly much more work needed to further develop this area and hopefully provide the required level of evidence for making dosing recommendations that will positively impact patient treatment. In this respect, there are positive signs that progress is being made. In the United States, plans are underway to conduct a prospective study to validate the recently proposed COG Chemotherapy Standardization Task Force recommendations for the use of dosing tables for infants to gradually transition from body weight to BSA-based dosing [32]. In the United Kingdom and the Netherlands, there are ongoing studies designed to investigate drug disposition in neonate and infant cancer patients, incorporating TDM and adaptive dosing approaches as appropriate, which have the potential to generate a wealth of data in this understudied patient population (https://www.isrctn.com/ISRCTN10139334 and https://www.trialregister.nl/trial/7527). Alongside the conduct of well-planned population pharmacokinetic studies in neonates and infants, the advancement of minimal sampling techniques for conducting such studies and the utility of physiologically-based pharmacokinetic model development to investigate physiological factors that may influence pharmacokinetics and evaluate contrasting dosing regimens in this patient population, there are clear indications that advancements in this field are gathering pace [[144], [145], [146], [147], [148]].

It is hoped that active research over the coming years will allow us to redefine dosing regimens for selected anticancer drugs as well as identify additional drugs that may benefit from adaptive dosing. In this way, we may be able to truly optimise dosing regimens in a patient population where pharmacokinetic parameters can be difficult to predict and may be rapidly changing with time. In the meantime, it is hoped that the pharmacological information collated in the present study acts as a temporary solution in providing a clinical tool to support dosing decisions in this challenging patient population.

## Funding

This work was supported in part by the 10.13039/501100000272National Institute for Health Research (NIHR) Research for Patient Benefit programme (PB-PG-1216-20032), 10.13039/501100000289Cancer Research UK (C9380/A25138) and the Experimental Cancer Medicine Centre Network (C9380/A25169). The views expressed are those of the authors and not necessarily those of the NIHR or the Department of Health and Social Care. The position of VC is funded by the Sir Bobby Robson Foundation.

## Conflict of interest statement

There are no competing interests to declare.
