# Supplementary material for: Clinical pharmacology of cytotoxic drugs in neonates and infants: Providing evidence-based dosing guidance
Source: Eur J Cancer. 2022 Mar;164:137–54. doi: 10.1016/j.ejca.2021.11.001 (PMC8914347; doi:10.1016/j.ejca.2021.11.001)
Supplement: Multimedia component 1 [file mmc1.docx]

**Supplemental Methods**

**Clinical pharmacology of cytotoxic drugs in neonates and infants: providing evidence-based dosing guidance**

A. Laura Nijstad, Shelby Barnett, Arief Lalmohamed, Inez M. Bérénos, Elizabeth Parke, Vickyanne Carruthers, Deborah A. Tweddle, Jordon Kong, C. Michel Zwaan, Alwin D.R. Huitema, Gareth J. Veal

1. **Methods**
   1. ***Literature search and study selection***

Studies were identified by searching the electronic Pubmed database using the following query:

((pharmacokinetic*[Title/Abstract]) AND ((***drug***[Title/Abstract]) OR (***drug synonym***[Title/Abstract]))
AND ((child*[Title/Abstract]) OR (pediatric*[Title/Abstract]) OR (infant*[Title/Abstract])))

Studies reporting pharmacokinetics of the chemotherapeutic agents of interest in infants and children were eligible for inclusion. The drugs of interest are presented in table S1 and eligibility criteria in table S2. The Pubmed results were subsequently screened by title, abstract and full text, respectively. Relevant articles that were found via references of identified studies were added to database.

*Table S1. Chemotherapeutic agents of interest*

| **Chemotherapeutic agent** | | |
| --- | --- | --- |
| Actinomycin D  Blinatumomab  Busulfan  Carboplatin  Cisplatin  Cyclophosphamide  Cytarabine  Daunorubicin  Dexamethasone  Dinutuximab | Doxorubicin Etoposide  Fludarabine  Gemtuzumab ozogamicin  Idarubicin  Ifosfamide  Irinotecan  Isotretinoin  Melphalan | Mercaptopurine  Methotrexate (high and low dose)  Mitoxantrone  Pegaspargase  Prednisolone  Temozolomide  Thioguanine  Topotecan  Vincristine |

*Table S2. Eligibility criteria for selection of relevant articles.*

| **Inclusion criteria** | **Exclusion criteria** |
| --- | --- |
| - Articles about the pharmacokinetics of the drug in children with cancer - Case reports on PK of the drug in children <4 years of age - Full text available - English manuscript | - Studies that did not include any patients below the age of 4 years - Studies concerning patients with impaired renal/hepatic function |

- 1. ***Data collection***

Two reviewers extracted the following data from the included studies in the database: PK methods (type of PK analysis, covariates for PK parameters), number of patients and infants included in the study, age and age related findings. Two reviewers checked the extracted data.

- 1. ***Levels of evidence***

Inspired by the Oxford Centre for Evidence-Based Medicine (CEBM) levels of evidence, five levels of evidence for PK studies were established^1,2^. The criteria for the levels of evidence are presented in table S3. The reviewers categorized the included studies from 1 to 5 for the level of evidence.

Table S3. Levels of evidence^1,2^

| **Level** | **Criteria** |
| --- | --- |
| 1 | Population PK model including infants |
| 2 | PK model including infants  Poor quality population PK model (effect of age not studied)  Population PK model without infants |
| 3 | Non compartmental PK study  Poor quality PK model (effect of age not studied)  PK model without infants |
| 4 | Case-report/-series |
| 5 | Mechanism-based reasoning  Expert opinion |

- 1. ***Grades of recommendation***

Grades of recommendations were subsequently established based on the grades of recommendation of the CEBM^1,2^, see table S4. The PK findings for all the individual drugs were summarized in table 4, and a grade for each chemotherapeutic agent was derived. Five reviewers individually graded all the drugs and disagreements were resolved by discussion between the reviewers. Thereafter the reviewers agreed on a recommended dose or dose adjustment for neonates and infants for every chemotherapeutic agent.

Table S4. Grades of recommendation^1,2^

| **Grade** | **Criteria** | **Clinical meaning** |
| --- | --- | --- |
| A | Convincing and consistent data of level 1 studies | Sufficient evidence available to recommend dosing in neonates/infants |
| B | Consistent level 2 or 3 studies or extrapolations from level 1 studies | Some evidence to guide dosing in neonates/infants available, but more information is needed |
| C | Level 4 studies or extrapolations from level 2 or 3 studies | Paediatric data available but no studies in neonates/infants or inconsistent data |
| D | Level 5 evidence or troublingly inconsistent or inconclusive studies of any level | No relevant data available |

**References**

1. Oxford Centre for Evidence-Based Medicine: OCEBM Levels of Evidence Working Group*, "The Oxford 2011 Levels of Evidence". Accessed via: <https://www.cebm.ox.ac.uk/resources/levels-of-evidence/ocebm-levels-of-evidence>
   * OCEBM Levels of Evidence Working Group = Jeremy Howick, Iain Chalmers (James Lind Library), Paul Glasziou, Trish Greenhalgh, Carl Heneghan, Alessandro Liberati, Ivan Moschetti, Bob Phillips, Hazel Thornton, Olive Goddard and Mary Hodgkinson
2. Oxford Centre for Evidence-Based Medicine: Levels of Evidence (March 2009). Accessed via: <https://www.cebm.ox.ac.uk/resources/levels-of-evidence/oxford-centre-for-evidence-based-medicine-levels-of-evidence-march-2009>
