# Supplementary material for: Clinical pharmacology of cytotoxic drugs in neonates and infants: Providing evidence-based dosing guidance
Source: Eur J Cancer. 2022 Mar;164:137–54. doi: 10.1016/j.ejca.2021.11.001 (PMC8914347; doi:10.1016/j.ejca.2021.11.001)
Supplement: Multimedia component 2 [file mmc2.docx]

**Supplemental Table S5**

**Clinical pharmacology of cytotoxic drugs in neonates and infants: providing evidence-based dosing guidance**

A. Laura Nijstad, Shelby Barnett, Arief Lalmohamed, Inez M. Bérénos, Elizabeth Parke, Vickyanne Carruthers, Deborah A. Tweddle, Jordon Kong, C. Michel Zwaan, Alwin D.R. Huitema, Gareth J. Veal

Table S5. Overview of PK publications

| **Author** | **Method** | **Number of patients/**  **infants (<1 yrs)** | **Age (yr),**  **median (range)** | **Age related findings** | **Level** | **Ref** |
| --- | --- | --- | --- | --- | --- | --- |
| ***Actinomycin D*** | | | | | | |
| Veal (2005) | Three compartment popPK model | 31 / 0 | 7 (1-20) | Effect of age was not studied. | 2 | ^1^ |
| Mondick (2008) | Three compartment popPK model  Allometric scaling for BW. | 33 / 0 | NS (1.6-20.3) | No age related effects independent of BW. | 2 | ^2^ |
| Edwards (2012) | Three compartment popPK model  Allometric scaling for BW.  Age was included as covariate on V1. | 36 / 0 | NS (1.6-20.3) | Age was included as covariate on V1, indicating a lower V1, corrected for BW, in older patients. | 2 | ^3^ |
| Hill (2014) | Three compartment popPK model  Allometric scaling for BW. | 117 / 9 | 4.6 (0.3-19.8) | Effect of age on PK not discussed. No effect of age on toxicity grade. | 1 | ^4^ |
| Balis (2017) | Mechanism-based development of dose bands based on BSA intervals | NA | NA | NA | 5 | ^5^ |
| Skolnik (2021) | Non-compartmental pharmacokinetics | 53 / 4 | NS (0.46-16.7) | Variability in t_1/2_, AUC and CL was high in children <1 years. The median AUC for children <1 years was approximately 50% lower than for older children (relatable to the more than twofold higher BSA-normalized dose in children ≥ 1 year. Dose did not appear to be related to AUC and CL corrected for BSA did not appear to be related to age. | 3 | ^6^ |
| ***Blinatumomab*** | | | | | | |
| Von Stackelberg (2016) | Non-compartmental pharmacokinetics | 49 / NS - Phase I  44 / NS - Phase II | 6 (<1-16) - Phase I  10.5 (<1-17) -Phase II | Pharmacokinetics consistent between age groups. | 3 | ^7^ |
| Clements (2020) | One compartment popPK model  BSA was included as covariate on CL. | 674 / >2 (Fig. 1b) | 41.0 (0.6-80) | No effect of age on BSA-normalized CL (L/h/m^2^). Dosing should be based on BSA in younger patients. | 1 | ^8^ |
| ***Busulfan*** | | | | | | |
| Dalle (2003) | Non-compartmental pharmacokinetics | 14 / 14 | 0.4 (0.06-1) | Cumulative dose of 16 mg/kg leads to increased exposure in infants compared to adults.  No correlation between age and AUC in the complete cohort. A trend for correlation between age and AUC in the female subgroup was seen. | 3 | ^9^ |
| Nguyen (2004) | One compartment popPK model | 24 / 3 | 6.0 (0.45-16.7), mean (range) | A log-linear relationship between BW and CL was demonstrated with no further age-dependency.  A novel dosing regimen (mg/kg dose adjusted to discrete weight categories) for a better AUC targeting was developed. | 1 | ^10^ |
| Tran (2004) | One compartment popPK model | 20 / 1 | 5.5 (0.8-14.9) | CL (mL/min/kg) was higher in children <6 years than in patients >6 years. | 2 | ^11^ |
| Oechtering (2005) | One compartment popPK model | 19 / 1 | 4 (0.9-17.3) | No effect of age on exposure. | 2 | ^12^ |
| Kletzel (2006) | Non-compartmental pharmacokinetics | 30 / 8 | 8 (0.11-16) | Effect of age was not studied. | 3 | ^13^ |
| Zwaveling (2006) | One compartment PK model | 18 / 1 | 7.0 (0.5-16) | CL (L/h/kg) proved to be dependent on age, whereas CL (L/h/m^2^) was age-independent. | 2 | ^14^ |
| Booth (2007) | One compartment popPK model  Allometric scaling for BW. | 24 / NS | 6.3 (0.25-16.7), mean (range) | No effect of age on CL after adjusting for BW. | 1 | ^15^ |
| Schechter (2007) | One compartment popPK model | 45 / 13 | 3.0 (0.25-16.2) | CL (mL/min/kg) was significantly higher in children <4 yrs. However, CL (mL/min/m^2^) was significantly lower in children <1 yrs and <4 yrs.  V (L/kg) was significantly higher in children <1 year or <4 years. | 2 | ^16^ |
| Nath (2008) | One compartment PK model | 40 / 7 | 3.2 (1.5-9.1) | Age correlated with CL (L/h) and CL (L/h/kg), but not with CL (L/h/m^2^). AUC did not correlate with age. | 2 | ^17^ |
| Kim (2009) | Non-compartmental pharmacokinetics | 21 / NS | 8 (0.25-18), mean (range) | CL (mL/min/kg) was significantly higher in children <4 yrs. | 3 | ^18^ |
| Wall (2009) | Non-compartmental pharmacokinetics | 24 / 3 | 3.3 (0.5-16.7) | No effect of age on CL (mL/min/kg). | 3 | ^19^ |
| Trame (2011) | One compartment popPK model  Allometric scaling for BW. | 94 / NS | 9.2 (0.4-18.8) | Effect of age was not described. | 2 | ^20^ |
| Bartelink (2012) | Two compartment popPK model  Allometric scaling for BW. | 245 / NS | 3.33 (0.1-26) | Non-linear relationship between BW and CL (L/h): An increase in BW in neonates results in a larger increase in CL than an increase in BW in older children or adults. | 1 | ^21^ |
| Bartelink (2012) | Two compartment popPK model  Allometric scaling for BW. | 403 / NS | 4.00 (0.1-35) | Non-linear relationship between BW and CL (L/h): An increase in BW in neonates results in a larger increase in CL than an increase in BW in older children or adults. | 1 | ^22^ |
| Michel (2012) | One compartment popPK model | 67 / 9 | 4.0 resp. 7.5 (autoSCT resp. alloSCT) (0.3-17.2) | Non-linear relationship between BW and clearance. | 1 | ^23^ |
| Paci (2012) | One compartment popPK model  Allometric scaling for BW. | 205 / NS | 2.5 (0.03-15) | The higher allometric exponent for CL accounted for a larger increase of CL in children <9 kg. For infants <9 kg, the model predicted a 2.4-fold increase in CL for a doubling in BW, whereas a 1.7-fold increase in CL was associated for the same BW growth in children ≥9 kg. | 1 | ^24^ |
| Veal (2012) | One compartment popPK model  Allometric scaling for BW. | 38 / NS | 3.6 (0.7-13.1), mean (range) for iv subgroup | Effect of age was not described. | 2 | ^25^ |
| Le Gall (2013) | Non-compartmental pharmacokinetics | 49 / 3 | 7.4 (0.2-18.4), mean (range) | CL (mL/min/kg) was significantly higher in children <4 yrs. | 3 | ^26^ |
| McCune (2013) | Non-compartmental pharmacokinetics, one or two compartment PK model  Allometric scaling for BW. | 729 / NS | 5.0 (0.1-20.0) | Non-linear relationship between age and CL (mL/min/kg). | 2 | ^27^ |
| Savic (2013) | One compartment popPK model  Allometric scaling for BW.  CL corrected for maturation. | 149 / NS (20 <0.5 yrs) | 0.94 (0.08-3.3) | CL increases by approximately 1.7-fold between 6 weeks and 2 years of life due to maturation. | 1 | ^28^ |
| Diestelhorst (2014) | One compartment popPK model  Allometric scaling for BW. | 82 / NS | NS (0.4-18.8) | Effect of age was not described. | 2 | ^29^ |
| McCune (2014) | Two compartment popPK model  Allometric scaling for BW.  CL corrected for maturation and sex  V1 and V2 corrected for sex | 1610 / 256 | 9.8 (0.1-66), mean (range) | The maturation of CL reaches 50% of adult values at 6 weeks after birth assuming a full-term gestational age of 40 weeks.  Size-standardized CL reaches 95% of adult values at 2.5 yrs. | 1 | ^30^ |
| Okamoto (2014) | One compartment popPK model  BW was included as covariate on CL and V. | 25 / 5 | 6 (0.4-17) | Effect of age was not described. | 2 | ^31^ |
| Long-Boyle (2015) | One compartment popPK model  Allometric scaling for BW.  CL corrected for maturation. | 90 / NS | 7 (0.1-24) | CL (adjusted for BW) increases up through 12 yrs and then begins to decline to adult levels. | 1 | ^32^ |
| Neely (2016) | One compartment popPK model  Allometric scaling for BW.  CL and V corrected for age. | 53 / NS | 7.8 (0.2-19), mean (range) | CL (adjusted for BW) increases up through ~7 yrs and then begins to decline to adult levels. | 1 | ^33^ |
| Nava (2018) | One compartment popPK model  Allometric scaling for BW.  CL corrected for maturation and GSTA1 diplotypes | 112 / 20 | 5.4 (0.1-20) | CL was corrected for maturation. | 2 | ^34^ |
| Alsultan (2020) | One compartment popPK model  Allometric scaling for BW. | 59 / NS | 6.1 (0.16-13), mean (range) | Effect of age was not described. | 2 | ^35^ |
| Marsit (2020) | One compartment popPK model  Allometric scaling for BW.  CL and V corrected for age. | 136 / NS | 6.6 (0.2-20.7), mean (range) | Age was included as covariates on all parameters. | 1 | ^36^ |
| Poinsignon (2020) | One compartment popPK model  Allometric scaling for BW.  CL and V were corrected for maturation. | 540 / 162 | 1.8 (0.02-24.1), mean (range) | All parameters were corrected for maturation. | 1 | ^37^ |
| Yuan (2021) | One compartment popPK model  CL corrected for BSA, GSTA1 diplotypes and ASAT. V corrected for BSA. | 69 / NS | 4.90 (0.50-15.18) | No effect of age after including BSA as covariate. | 2 | ^38^ |
| Neroutsos (2021) | Non-compartmental pharmacokinetics | 76 / NS (12 <2 years) | 6.5 (0.5-19) | Effect of age was not studied. | 3 | ^39^ |
| Ben Hassine (2021) | Two compartment popPK model  Postmenstrual age dependent allometric scaling of BW on CL.  CL corrected for day of therapy, GSTA1 diplotypes and regimen including fludarabine. | 302 / NS | 5.2 (0.1-20.1) | CL was corrected for maturation. | 1 | ^40^ |
| ***Carboplatin*** | | | | | | |
| Riccardi (1992) | Case series, two compartment PK model (total and free platinum) | 5 / 0 | 6 (1.5-7) | Effect of age was not studied. | 4 | ^41^ |
| Madden (1992) | Two compartment popPK model (total and free platinum) | 18 / 0 | 7.7 (2.9-23) | No effect of age on CL after normalization to BW | 2 | ^42^ |
| Newell (1993) | Two compartment PK model (total and free platinum) | 22 / 4 | 3.8 (0.3-15.8) | Effect of age was not studied only the impact of EDTA CL on dosing. Significant correlation between EDTA CL and carboplatin CL. | 3 | ^43^ |
| Riccardi (1994) | Two compartment PK model (free platinum) | 35 / NS | 7 (0.8-17) | Effect of age was not studied but PK parameters comparable to those reported in adults. | 3 | ^44^ |
| Peng (1995) | Two compartment popPK model (total and free platinum) | 23 / 3 | 3.7 (0.08-18.4) | Effect of age was not studied. | 2 | ^45^ |
| Chatelut (1996) | Two compartment popPK model (free platinum)  CL corrected for BW, creatinine and nephrectomy | 57 / NS | 5 (0.17-18) | No additional effect of age on PK parameters that was not explained by BW or BSA. | 1 | ^46^ |
| Tonda (1996) | One compartment popPK model (free platinum) | 21 / 4 | 1.7 (0.2-4.2) | Median CL (mL/min/m^2^) in infants lower than median for older patients. | 1 | ^47^ |
| Doz (1998) | Two compartment popPK model (free platinum) | 15 / 1  16 / 1 | 5.0 (0.9-10.7)  4.6 (0.75-17.5) | Effect of age was not studied. | 2 | ^48^ |
| Thomas (2000) | Two compartment popPK model (free platinum) | 38 / NS | 3.5 (0.4-16.3) | No effect of age on dosing method (renal function vs BSA dosing). | 1 | ^49^ |
| Patoux (2001) | Two compartment popPK model (free platinum)  Serum creatinine was identified as the most significant covariate on CL. | 117 / NS | 6 (0.1-18.4) | No effect of age on CL relative to other covariates tested. | 1 | ^50^ |
| Rubie (2003) | Two compartment popPK model (free platinum) | 12 / 0 | 3.4 (2.8-17.3) | Effect of age was not studied. | 2 | ^51^ |
| Kangarloo (2004) | Non-compartmental pharmacokinetics and PK model (total and free platinum) | 10 / 2 | 11 (0.25-16) | Effect of age was not studied. | 3 | ^52^ |
| Veal (2007) | Two compartment popPK model (free platinum) | 28 / 0 | 11.7 (1-21) | Effect of age on PK was not studied. No age effect on platinum adduct formation. | 2 | ^53^ |
| Levy (2009) | Non-compartmental pharmacokinetics (free platinum) | 28 / 0 | 8.5 (1-21) | Effect of age was not studied. | 3 | ^54^ |
| Picton (2009) | Case series, two compartment PK model (free platinum) | 1 / 1 | Gest. age 32 weeks | CL (mL/min) increased by 2-fold over 7 weeks. | 4 | ^55^ |
| Veal (2010) | Two compartment popPK model (free platinum)  BW was included as covariate on PK parameters. | 19 / 16 | 0.8 (0.2-1.2) | CL (mL/min/kg) higher in infants <12kg compared to children >12kg. | 1 | ^56^ |
| Veal (2015) | Therapeutic drug monitoring in preterm and full-term neonates. CL and AUC were calculated using the PK model of Veal (2007) and Peng (1995). | 9 / 9 | 3 weeks (0.4-24 weeks) (gest. age 40 weeks (35-52 weeks)) | CL (mL/min and mL/min/kg) increased with increasing age (normalized to full-term gestation). | 1 | ^57^ |
| Veal (2016) | Case series, non-compartmental pharmacokinetics (free platinum) | 1 / 1 | 8 weeks (gest. age 40 weeks) | CL normalized for BW comparable with reported values. | 4 | ^58^ |
| Balis (2017) | Mechanism-based development of dose bands based on BSA intervals | NA | NA | NA | 5 | ^5^ |
| Duong (2019) | Two compartment popPK model (free platinum)  Allometric scaling for BW. | 46 / 0 | 3.5 (1.7-8.3) | No effect of age on the PK parameters after including BW into the model. | 2 | ^59^ |
| Hawley (2019) | Case series, non-compartmental pharmacokinetics (free platinum) | 1 / 1 | 5 weeks (gest. age 40 weeks) | Effect of age not discussed, but under standard dosing regimen patient would have been under-dosed | 4 | ^60^ |
| Hong (2020) | Non-compartmental pharmacokinetics and one compartment popPK model (total platinum)  eGFR was included as covariate on CL. | 25 / 0 | 6 (1-17) | Effect of age was not studied. | 2 | ^61^ |
| ***Cisplatin*** | | | | | | |
| Crom (1981) | Two compartment PK model (total platinum) and non-compartmental pharmacokinetics (free platinum) | 28 / 0 | 11.4 (1.7-20.5) | The elimination rate constant of total platinum was larger in older children (more rapid elimination). | 3 | ^62^ |
| Dominici (1989) | Non-compartmental pharmacokinetics (total and free platinum) | 14 / NS | NS (0.8-13) | Effect of age was not studied. | 3 | ^63^ |
| Murakami (1990) | One compartment PK model (free platinum) | 6 / 0 | 9.4 (1.7-15.7) | Younger patients showed a lower CL and a higher V. | 3 | ^64^ |
| Peng (1997) | Non-compartmental pharmacokinetics and one compartment PK model (free platinum) | 21 / 1 | 9.1 (0.5-19.3) | Effect of age was not studied. | 3 | ^65^ |
| Balis (2017) | Mechanism-based development of dose bands based on BSA intervals | NA | NA | NA | 5 | ^5^ |
| Thomas (2018) | Case report, non-compartmental pharmacokinetics | 1 / 1 | 2 weeks (gest. age 37 weeks) | CL was correlated to age and BW. CL (mL/min/kg) increased with increasing age. | 4 | ^66^ |
| ***Cyclophosphamide*** | | | | | | |
| Juma (1984) | Non-compartmental pharmacokinetics  Kenyan patients | 8 / 0 | NS (1-4) | Effect of age not studied in this patient group. However, CL (mL/h/kg) values here are higher than those reported in adults. | 3 | ^67^ |
| Tasso (1992) | Non-compartmental pharmacokinetics | 9 / 2 | 4.3 (0.7-16.8) | Effect of age not studied in this patient group. However, CL (L/h/m^2^) values here are higher than those reported in adults. | 3 | ^68^ |
| Yule (1996) | One compartment popPK model | 38 / 5 | 4 (0.2-18) | No additional effect of age on PK parameters that was not explained by BSA. | 1 | ^69^ |
| Yule (1997) | PopPK model (compartments not described) | 14 / 1 | 8.5 (0.7-17) | Effect of age on PK was not studied. | 2 | ^70^ |
| Yule (2001) | One compartment popPK model | 13 / 0 | 4 (2-17) | No effect of age on PK parameters. | 2 | ^71^ |
| Yule (2004) | One compartment popPK model | 36 / 0 | 8 (2-16) | Effect of age on PK was not studied. | 2 | ^72^ |
| McCune (2009) | Multicompartment popPK model (one compartment for cyclophosphamide, three compartments for metabolites) | 22 / 0 | 3.16 (1.3-9.37) | Cyclophosphamide AUC associated with patient age and BSA. | 2 | ^73^ |
| Balasubramanian (2012) | Two compartment popPK model (cyclophosphamide)  One compartment popPK model (metabolites)  BW, age and CYP2C19 polymorphism were included as covariates for CL | 55 / 0 | 7.3 (2-14) | Decrease in CL (L/h/kg) with patient age. | 2 | ^74^ |
| Navid (2013) | Two compartment popPK model | 19 / 0 | 9.2 (1.2-24.5) | Effect of age was not studied. | 2 | ^75^ |
| Veal (2016) | Two compartment popPK model  Allometric scaling for BSA.  CYP2B6 polymorphism and eGFR correlated with CL. | 49 / 0 | 11.7 (3.5-18.7) | No effect of age on CL (L/h/m^2^). | 2 | ^76^ |
| Balis (2017) | Mechanism-based development of dose bands based on BSA intervals | NA | NA | NA | 5 | ^5^ |
| Campagne (2020) | Two compartment popPK model (cyclophosphamide)  One compartment popPK model (metabolites)  Age, phenobarbital treatment and CYP2B6 polymorphism correlated with PK. | 171 / 42 | 1.8 (0.07-4.9) | Infants had higher exposures of the active metabolite compared to older children. Suggested to decrease dose by 20% in young infants to achieve similar exposures to older children. | 1 | ^77^ |
| Barnett (2021) | Two compartment popPK model  Allometric scaling for BSA.  CYP2B6 polymorphism and eGFR were included as covariates for CL. | 25 / 12 | 1 (0.33-2) | No effect of age on PK within this study. However, CL (mL/min/m^2^) of cyclophosphamide and AUC of metabolites in children <2 years old significantly higher than those reported in older children. | 1 | ^78^ |
| ***Cytarabine (ARA-C)*** | | | | | | |
| Avramis (1987) | Non-compartmental pharmacokinetics (ARA-C and ARA-U) | 20 / 0 | 3 (1.5-19) | AUC (ARA-C) higher in children compared to adults.  Patients <2 yrs had higher ARA-C concentrations than older patients | 3 | ^79^ |
| Avramis (1989) | Two compartment PK model (ARA-C)  One compartment PK model (ARA-U)  Age and BSA were included as covariates on all parameters. | 8 / 1 | 6.5 (0.58-16) | Age was included as covariate on all parameters. | 3 | ^80^ |
| McLeod (1992) | One compartment PK model | 67 / 3 | NS (0.64-19) | CL (mL/min/m^2^) is not different in infants. | 2 | ^81^ |
| Periclou (1996) | Two compartment popPK model (ARA-C)  One compartment popPK model (ARA-U)  Age and BSA were included as covariates on all parameters. | 52 / NS | NS (0.2-19) | All parameters increase with increasing age and BSA.  Conversion of ARA-C to ARA-U is age dependent (lower rate of conversion in infants). | 1 | ^82^ |
| Avramis (1998) | Non-compartmental pharmacokinetics (ARA-C) | 20 / 0 | 8 (1-19) | Effect of age was not studied. | 3 | ^83^ |
| Evans (1998) | One compartment PK model | 182 / 8 | NS (0.3-18.8) | Effect of age was not studied. | 3 | ^84^ |
| Ozkaynak (1998) | Non-compartmental pharmacokinetics (ARA-C and ARA-U) | 13 / 0 | 7 (1-17) | Effect of age was not studied. | 3 | ^85^ |
| ***Daunorubicin*** | | | | | | |
| Hempel (2010) | Two compartment popPK model | 33 / 20 | NS (0.05-18.8) | No correlation between PK parameters, dose normalized for BSA, and age. | 1 | ^86^ |
| Thompson (2014) | Two compartment popPK model | 98 / NS | 12 (0.5-20.4) | No correlation between PK parameters and age or body composition. | 1 | ^87^ |
| Balis (2017) | Mechanism-based development of dose bands based on BSA intervals | NA | NA | NA | 5 | ^5^ |
| ***Dexamethasone*** | | | | | | |
| Spektor (2008) | Non-compartmental pharmacokinetics | 25 / 0 | 4 (1-14) | Effect of age was not studied. But t_1/2_ comparable to data reported in adults. | 3 | ^88^ |
| Yang (2008) | One compartment popPK model | 214 / 0 | NS (1-18.8) | Negative correlation between patient age and CL/F (normalized to BSA). Higher CL in younger patients. | 2 | ^89^ |
| Kawedia (2012) | Not stated | 339 / 0 | NS | CL (L/h/m^2^) higher in patients less than 10 years old | 3 | ^90^ |
| Inaba (2018) | One compartment popPK model | 409 / 0 | NS (1-18) | Effect of age on PK was not studied. | 2 | ^91^ |
| Jackson (2019) | One compartment popPK model  Allometric scaling of CL for BW. | 161 / 0 | NS (1.3-18.7) | No effect of age on PK. | 2 | ^92^ |
| ***Dinutuximab*** | | | | | | |
| Uttenreuther-Fischer (1995) | Two compartment PK model | 10 / 0 | 6.4 (2.1-11.4) | Effect of age was not studied. t_1/2_ values lower than those reported in adults | 3 | ^93^ |
| Ladenstein (2013) | Non-compartmental pharmacokinetics | 16 / 0 | 7.6 (3.8-17.3) | Effect of age was not studied. | 3 | ^94^ |
| Desai (2014) | Non-compartmental pharmacokinetics and Two compartment PK model | 14 / 0 | 4.3 (1.2-7.3) | CL normalized to BW shows a negative correlation with age. Younger children exhibit higher CL values. | 3 | ^95^ |
| Marachelian (2016) | Two compartment popPK model  BW included as an allometric covariate on CL and V in the model. | 28 / 0 | 4 (2-7) Arm 1  4 (1-9) Arm 2, mean (range) | Effect of age not studied. | 2 | ^96^ |
| ***Doxorubicin*** | | | | | | |
| McLeod (1992) | Non-compartmental pharmacokinetics (doxorubicin) | 60 / 4 | NS (0.17-20) | No difference in CL (mL/min/kg) between infants and older children, but there was a trend toward a lower CL (mL/min/m^2^) in infants compared to older children. | 3 | ^81^ |
| Eksborg (2000) | Non-compartmental pharmacokinetics (doxorubicin) | 31 / NS | 5.4 (0.73-15.3) | No correlation between age and Cmax after dose normalization for body size. | 3 | ^97^ |
| Frost (2002) | Non-compartmental pharmacokinetics (doxorubicin and doxorubicinol) | 112 / 5 | 4.7 (for pts >1 yr) (0.3-17.3) | Children 4-6 yrs had the highest dose normalized steady state plasma concentration, followed by children 2-4 yrs, younger and older pts showed similar plasma concentrations. | 3 | ^98^ |
| Hempel (2002) | Non-compartmental pharmacokinetics (doxorubicin) | 27 / 0 | 4.13 (1.56-19.99) | Age had no effect on Cmax | 3 | ^99^ |
| Palle (2006) | Non-compartmental pharmacokinetics (doxorubicin) | 37 / 1 | 9.2 (0.63-17.7) | No sign differences in CL (mL/min/m^2^) between children <2 yrs and older children | 3 | ^100^ |
| Thompson (2009) | Three compartment popPK model (doxorubicin)  One compartment popPK (doxorubicinol)  Allometric scaling for BSA. | 22 / 0 | 15.0 (3.3-21.5) | No correlation between PK parameters and age after adjusting for BSA. | 2 | ^101^ |
| Köntny (2013) | Three compartment popPK model (doxorubicin)  One compartment popPK (doxorubicinol)  Allometric scaling for BSA. | 82 / 0 | 21 (3-73) | After adjusting for BSA: No correlation between CL of doxorubicin and doxorubicinol and age. A trend towards a smaller V1 and a larger Vm in younger patients was observed. | 2 | ^102^ |
| Völler (2015) | Three compartment popPK model (doxorubicin)  One compartment popPK (doxorubicinol)  Allometric scaling for BSA.  Age was included as covariate on CL of doxorubicin. | 94 / 4 | 5.32 (0.2-17.7) | Age was included as covariate on CL of doxorubicin, indicating a lower CL, corrected for BSA, in younger patients. | 1 | ^103^ |
| Krischke (2016) | Three compartment popPK model (doxorubicin)  One compartment popPK (doxorubicinol)  Allometric scaling for BSA.  Age was included as covariate on CL of doxorubicin. | 101 / NS | 5.3 (0.2-17.7) | Age dependence of CL of doxorubicin: <3 years having a statistically significant lower CL than older children after correcting for BSA. | 1 | ^104^ |
| Balis (2017) | Mechanism-based development of dose bands based on BSA intervals | NA | NA | NA | 5 | ^5^ |
| Kunarajah (2017) | Three compartment popPK model (doxorubicin)  One compartment popPK (doxorubicinol)  Allometric scaling for BSA.  Age was included as covariate on CL of doxorubicin. | 17 / 0 | 7.50 (3.4-14.7) | Age was included as covariate on CL, indicating a lower CL, corrected for BSA, in younger patients. | 2 | ^105^ |
| Siebel (2020) | Pharmacokinetic simulations using the popPK model of Völler (2015). | 94 / 4 | 5.32 (0.2-17.7) | Conclusions: treatment strategies for young children should adapt both the dose (based on age and BSA) and the duration of infusion. | 5 | ^106^ |
| ***Etoposide*** | | | | | | |
| Evans (1982) | Two compartment PK model | 9 / NS | 10 (0.25-18) | CL comparable with adults. | 2 | ^107^ |
| McLeod (1992) | Two compartment PK model | 25 / 2 | NS (0.5-18) | CL (mL/min/kg) was lower in infants compared to older children. However, no difference in CL (mL/min/m^2^). | 2 | ^81^ |
| Lowis (1993) | Two compartment PK model | 33 / NS | 4.8 (0.4-16.1) | Effect of age was not studied. | 2 | ^108^ |
| Rodman (1994) | Two compartment PK model | 22 / 0 | 6.7 (1.6-23.9) | No effect of age on CL after adjusting for body size. | 3 | ^109^ |
| Boos (1995) | Evaluation of steady state plasma concentrations | 40 / 11 | 2.4 (0.25-28) | Dosing in mg/kg let to significantly lower Css values than dosing in mg/m^2^.  No effect of age on CL (mL/min/m^2^ and mL/min/kg). | 3 | ^110^ |
| Würthwein (1999) | Two compartment popPK model | 18 / 1 | 10.5 (0.8-17) | Effect of age was not studied. | 2 | ^111^ |
| Eksborg (2000) | One or two compartment PK model | 16 / 1 | 8.3 (0.3-22) | AUC normalized to BSA was not age dependent. t_½_ was not age dependent. | 2 | ^112^ |
| Lacayo (2002) | Two compartment popPK model  Pharmacokinetic interaction with cyclosporine | 38 / NS | NS (0.7-17) | Effect of age was not studied. | 2 | ^113^ |
| Würthwein (2002) | Three compartment popPK model | 31 / 1 | 8.0 (0.8-23.7) | No correlation between CL (mL/min/m^2^) and age. A significant correlation between CL (mL/min/kg) and age. | 1 | ^114^ |
| Kato (2003) | One compartment PK model | 18 / 1 | 7.4 (0.3-18.0, mean (range) | No effect of age on t_½_, V or AUC. | 2 | ^115^ |
| Palle (2006) | Non-compartmental pharmacokinetics | 45 / 2 | 10.3 (0.5-17.7) | No effect of age on CL (mL/min/m^2^). | 3 | ^116^ |
| Veal (2010) | Two compartment popPK model  Allometric scaling for BW | 11 / 7 | 0.8 (0.2-1.2) | No effect of age on CL after including BW into the covariate model. | 1 | ^56^ |
| Urien (2011) | Two compartment popPK model  Allometric scaling for BW | 67 / 8 | 3.5 (0.3-16.7) | No effect of age on CL (using a sigmoid Emax model relating the post-menstrual age) after including BW into the model. | 1 | ^117^ |
| Baheti (2013) | One compartment popPK model  Allometric scaling for BW | 26 / 0 | 8.5 (2-19), mean (range) | Including age as covariate on CL and V did significantly reduce the objective function during stepwise forward addition process, but was not significant when backward elimination was performed. | 1 | ^118^ |
| Veal (2016) | Case series, non-compartmental pharmacokinetics | 1 / 1 | 8 weeks (gest. age 40 weeks) | CL and AUC comparable with reported values. | 4 | ^119^ |
| Balis (2017) | Mechanism-based development of dose bands based on BSA intervals | NA | NA | NA | 5 | ^5^ |
| Duong (2019) | Two compartment popPK model  Allometric scaling for BW. | 51 / 0 | 3.5 (1.7-8.3) | No effect of age on the PK parameters after including BW into the covariate model. | 2 | ^59^ |
| ***Fludarabine*** | | | | | | |
| Ivaturi (2017) | Two compartment popPK model  Allometric scaling for BW.  eGFR was included as covariate on CL. | 133 / NS | 5 (0.2-17.9) | No effect of age on CL, normalized to BW. | 1 | ^120^ |
| Chung (2019) | Two compartment popPK model  Allometric scaling for BSA.  eGFR was included as covariate on CL. | 43 / 0 | 11.8 (1.3-18.5) | No effect of age on CL, normalized to BSA. | 2 | ^121^ |
| Langenhorst (2019) | Three compartment popPK model  Allometric scaling for BW.  eGFR was included as covariate on CL. | 258 / NS | 18 (0.3-74) | After accounting for covariates, no size-independent effect of age was identified. | 1 | ^122^ |
| ***Gemtuzumab ozogamicin*** | | | | | | |
| Buckwalter (2004) | Non-compartmental pharmacokinetics | 29 / 0 | 9.6 (1-16), mean (range) | No statistical difference in PK parameters across age groups | 3 | ^123^ |
| Masters (2018) | Two compartment popPK model  Allometric scaling for BW. | 29 / 0 | 12 (1.2-16.9) | No age related effects independent of BW. | 2 | ^124^ |
| ***Idarubicin*** | | | | | | |
| Tan (1987) | Non-compartmental pharmacokinetics (idarubicin, idarubicinol) | 7 / 0 | NS (1-19) | Effect of age was not studied. | 3 | ^125^ |
| Reid (1990) | Non-compartmental pharmacokinetics (idarubicin, idarubicinol) | 21 / 0 | NS (1-21) | No effect of age on PK parameters of idarubicin or idarubicinol | 3 | ^126^ |
| Dreyer (2003) | Non-compartmental pharmacokinetics (idarubicin, idarubicinol) | 14 / 0 | 7.2 (2.2-19) | Effect of age was not studied. | 3 | ^127^ |
| ***Ifosfamide*** | | | | | | |
| Boddy (1993) | Non-compartmental pharmacokinetics | 16 / 0 | 4 (1-17) | No additional effect of age on PK parameters that was not explained by BW/BSA | 3 | ^128^ |
| Prasad (1994) | Non-compartmental pharmacokinetics | 5 / 0 | 14 (3-15) | Effect of age was not studied. | 3 | ^129^ |
| Boddy (1996) | Non-compartmental pharmacokinetics | 11 / 0 | NS (1-16) | Positive correlation between patient age and exposure | 3 | ^130^ |
| Kerbusch (2001) | One compartment popPK model | 32 / 0 | NS (1-18) | Effect of age was not studied. | 2 | ^131^ |
| Willits (2005) | Non-compartmental pharmacokinetics | 19 / 0 | 10 (1-19) | Effect of age on PK parameters not studied. No correlation between age and DNA damage | 3 | ^132^ |
| Balis (2017) | Mechanism-based development of dose bands based on BSA intervals | NA | NA | NA | 5 | ^5^ |
| ***Irinotecan*** | | | | | | |
| Crews (2002) | PopPK model (irinotecan and metabolites, compartments not described) | 31 / 0 | NS (3-21) | Effect of age was not studied. | 2 | ^133^ |
| Gajjar (2003) | Non-compartmental pharmacokinetics | 35 / 0 | NS (3-21) | Effect of age was not studied. | 3 | ^134^ |
| Vassal (2003) | Non-compartmental pharmacokinetics | 77 / NS | 8 (0.9-18.6) | Effect of age was not studied. But CL values (L/h/m^2^) in these children were higher than those reported in adults | 3 | ^135^ |
| Wagner (2004) | One compartment popPK model (additional compartments for metabolites) | 12 / 0 | 12.5 (1-23) | Effect of age was not studied. | 2 | ^136^ |
| Bomgaars (2006) | Non-compartmental pharmacokinetics | 9 / 0 (Stratum 1)  9 / 0 (Stratum 2) | 11 (4-17) S1  6 (2-15) S2 | Effect of age was not studied. | 3 | ^137^ |
| Furman (2006) | One compartment popPK model (additional compartments for metabolites) (after oral administration) | 39 / 0 | 10 (3-19) | Effect of age was not studied. | 2 | ^138^ |
| Rodriguez-Galindo (2006) | One compartment popPK model (additional compartments for metabolites) | 11 / 0 | 10 (3-19) | Effect of age was not studied. Comparable PK to children in other studies. | 2 | ^139^ |
| Bomgaars (2007) | Non-compartmental pharmacokinetics | 79 / 0 | 10 (2-23) | Effect of age was not studied. | 3 | ^140^ |
| Stewart (2007) | Multicompartment PopPK model (after i.v. and oral administration) | 74 / 0 | 10.4 (3.2-21.6) | Effect of age was not studied. | 2 | ^141^ |
| Thompson (2008) | Two compartment popPK model (additional compartments for metabolites)  Allometric scaling for BW.  Age and bilirubin included as covariates on SN-38 CL | 82 / 0 | NS (1-21) | Age and bilirubin significant covariates in SN-38 CL. CL (L/h) of SN-38 greater in patients <10 years old | 2 | ^142^ |
| Furman (2009) | Two compartment popPK model (additional compartments for metabolites) | 29 / 0 | 9 (1-21) | Effect of age was not studied. | 2 | ^143^ |
| Levy (2009) | Non-compartmental pharmacokinetics | 28 / 0 | 8.5 (1-21) | Effect of age was not studied. | 3 | ^54^ |
| Balis (2017) | Mechanism-based development of dose bands based on BSA intervals | NA | NA | NA | 5 | ^5^ |
| Jannier (2020) | Non-compartmental pharmacokinetics | 42 / 0 | 10.5 (2-18) | Effect of age was not studied. | 3 | ^144^ |
| ***Isotretinoin*** | | | | | | |
| Villablanca (1995) | Non-compartmental pharmacokinetics | 51 / 0 | 4 (2-12) | Effect of age was not studied. | 3 | ^145^ |
| Khan (1996) | One compartment PK model | 31 / 0 | 4 (2-12) | Effect of age could not be determined due to age distribution in patient cohort. | 3 | ^146^ |
| Veal (2007) | One compartment popPK model | 29 / 0 | 3.2 (1.1-18.7) | Higher weight and age associated with higher exposures. | 2 | ^147^ |
| Veal (2013) | One compartment popPK model | 103 / 10 | 4.3 (0.8-20.5) | No effect of age on PK parameters. | 1 | ^148^ |
| Gota (2016) | Non-compartmental pharmacokinetics | 35 / 0 | 5 (1-13) | No effect of age on exposure. | 3 | ^149^ |
| Balis (2017) | Mechanism-based development of dose bands based on BSA intervals | NA | NA | NA | 5 | ^5^ |
| Veal (2021) | Two compartment popPK model  Allometric scaling for BW. | 20 / 0 | 4.3 (1-1.66) | No additional effect of age on PK parameters that was not explained by BW. | 2 | ^150^ |
| ***Melphalan*** | | | | | | |
| Taha (1983) | One compartment PK model | 10 / 0 | 5.5 (2.5-16) | No effect of age on PK parameters | 3 | ^151^ |
| Ninane (1985) | Two compartment PK model | 9 / 1 | 4.1 (1.1-10) | Effect of age was not studied. | 3 | ^152^ |
| Ardiet (1986) | Two compartment PK model | 26 / 0 | 12.2 (1.3-57) | No difference in PK between adults in children. But a trend towards lower exposures in children due to shorter half-life. | 3 | ^153^ |
| Gouyette (1986) | Two compartment PK model | 20 / 0 | 4.4 (1.8-14) | Effect of age was not studied. | 3 | ^154^ |
| Horowitz (1988) | Non-compartmental pharmacokinetics | 26 / 1 | 11 (<1-21) | Effect of age was not studied. | 3 | ^155^ |
| Vassal (2001) | Two compartment popPK model | 21 / NS | 4.1 (0.7-14.2) | Effect of age on PK not studied. Correlation between age and neutropenia/platelet recovery. Younger age associated with prolonged neutropenia and delayed platelet recovery. | 2 | ^156^ |
| Nath (2005) | Non-compartmental pharmacokinetics | 52 / NS | 5.6 (0.3-18) | No additional effect of age on PK parameters that was not explained by BW. | 3 | ^157^ |
| Nath (2007) | Two compartment popPK model  BW, eGFR and previous carboplatin treatment were included as covariate on CL. BW was included as covariate on V. | 59 / NS | NS (0.3-17.6) | No effect of age on PK parameters. BW, creatinine CL important covariates for CL and BW for V. | 1 | ^158^ |
| Schaiquevich (2012) | Two compartment popPK model  BW was included as covariate on the PK parameters. | 17 / NS | 1.8 (0.6-6.2) | Inter-individual variability partly explained by age, BW and BSA. | 1 | ^159^ |
| Taich (2014) | Two compartment popPK model  The dose was normalized to BW. | 21 / NS | 1.7 (0.5-6.2) | No additional effect of age on PK parameters that was not explained by BW. | 1 | ^160^ |
| Mizuno (2018) | Two compartment popPK model  Allometric scaling for BW.  eGFR was included as covariate on CL. | 5 / 0 | 5.6 (1.5-16.5) | No effect of age on PK parameters. | 2 | ^161^ |
| Duong (2019) | Two compartment popPK model  Allometric scaling for BW. | 51 / 0 | 3.5 (1.7-8.3) | No effect of age on the PK parameters after including BW into the covariate model. | 2 | ^59^ |
| Zhao (2021) | Non-compartmental pharmacokinetics | 5 / 0 | 5.6 (1.5-16.8) | Effect of age was not studied. | 3 | ^162^ |
| ***Mercaptopurine (6-MP)*** | | | | | | |
| Lennard (1986) | Non-compartmental pharmacokinetics (plasma 6-MP, RBC 6-TGN) | 19 / 0 | 6.5 (3-16), mean (range) | No effect of age on any of the PK parameters was found. | 3 | ^163^ |
| Sulh (1986) | Non-compartmental pharmacokinetics (plasma 6-MP) | 20 / 0 | 7.5 (2-16), mean (range) | No effect of age on AUC or elimination half-life. | 3 | ^164^ |
| Lennard (1989) | Non-compartmental pharmacokinetics (RBC 6-TGN) | 120 / 0 | 5.3 (2-17) | No effect of age on the 6-TGN effect was found. | 3 | ^165^ |
| Koren (1990) | Non-compartmental pharmacokinetics (plasma 6-MP) | 23 / 0 | Means were 3.9 and 4.3 for two groups. Range NS. | Effect of age was not studied. | 3 | ^166^ |
| Lennard (1990) | Non-compartmental pharmacokinetics (RBC 6-TGN) | 95 / 0 | 5.3 (2-17) | No effect of age on the 6-TGN concentration was found. | 3 | ^167^ |
| Kato (1991) | Non-compartmental pharmacokinetics (plasma 6-MP) | 8 / 0 | 7.7 (3.6-15.1), mean (range) | No effect of age on AUC was found. | 3 | ^168^ |
| Zuccaro (1991) | Non-compartmental pharmacokinetics (plasma 6-MP) | 18 / 0 | 6.9 (3-15), mean (range) | Effect of age was not studied. | 3 | ^169^ |
| McLeod (1992) | Non-compartmental pharmacokinetics (RBC 6-TGN) | 110 / 3 | NS (0.58-19) | No difference in RBC 6-TGN concentrations between infants and older children. | 3 | ^81^ |
| Welch (1997) | Non-compartmental pharmacokinetics (RBC 6-MMP, RBC 6-TGN) | 7 / 0 | 4 (2-6) | Effect of age was not studied. | 3 | ^170^ |
| Balis (1998) | Non-compartmental pharmacokinetics (plasma 6-MP, RBC 6-TGN) | 89 / 0 | 4.6 (1.1-17.3) | No effect of age on CL (mL/min/m^2^) of 6-MP or RBC 6-TGN levels. | 3 | ^171^ |
| Erb (1998) | Non-compartmental pharmacokinetics (RBC 6-TGN) | 32 / 0 | NS (1-18) | Effect of age was not studied. | 3 | ^172^ |
| Mawatari (2001) | Non-compartmental pharmacokinetics (RBC 6-TGN) | 12 / 0 | 7 (2-13) | Effect of age was not studied. | 3 | ^173^ |
| Bell (2004) | Non-compartmental pharmacokinetics (RBC 6-MMP, RBC 6-TGN) | 226 / NS | 4.4 (3.1-6.8), median (quartiles) | No effect of age on metabolite levels. | 3 | ^174^ |
| Hawwa (2008) | One compartment popPK model (RBC 6-MMP, RBC 6-TGN)  TPMT mutation was included as covariate on formation of the 6-TGN metabolite  BSA was included as covariate on CL of 6-TGN. | 19 / 0 | 10 (3-17) | No effect of age on the PK parameters. | 2 | ^175^ |
| Hanff (2013) | Non-compartmental pharmacokinetics (RBC 6-MMP, RBC 6-TGN) | 20 / 0 | 4.1 resp 4.3 (1.9-14.6) | Effect of age was not studied. | 3 | ^176^ |
| Larsen (2020) | Non-compartmental pharmacokinetics (plasma 6-MP) | 12 / 0 | 5 (3.75-9.25), median (quartiles) | The relative performance of two formulations (tablet vs. liquid) with respect to dose normalized AUC was not associated with age. | 3 | ^177^ |
| ***Methotrexate (MTX) (low dose)*** | | | | | | |
| Pinkerton (1982) | Non-compartmental pharmacokinetics (MTX p.o. and i.v., plasma MTX concentrations) | 28 / 0 | NS (3-16) | No effect of age on absorption of MTX. | 3 | ^178^ |
| Sonneveld (1986) | Non-compartmental pharmacokinetics (MTX p.o., plasma MTX concentrations) | 19 / 0 | 6.3 (3-14), mean (range) | Effect of age was not studied. | 3 | ^179^ |
| Pearson (1987) | Non-compartmental pharmacokinetics (MTX p.o. and i.m., plasma MTX concentrations) | 127 / 0 | NS (1-14) | No effect of age on absorption or CL (L/h/m^2^) of MTX. | 3 | ^180^ |
| Balis (1988) | Non-compartmental pharmacokinetics (MTX p.o. and s.c., plasma MTX concentrations) | 8 / 0 | 8 (3-19) | Effect of age was not studied. | 3 | ^181^ |
| Koren (1989) | Non-compartmental pharmacokinetics (MTX p.o., plasma MTX concentrations) | 16 / 0 | 6.26 (3.75-16.75), mean (range) | A weak correlation between AUC and age was observed. | 3 | ^182^ |
| Skoglund (1994) | Non-compartmental pharmacokinetics (MTX p.o., plasma MTX concentrations) | 17 / 0 | 7.3 (3-12), mean (range) | Effect of age was not studied. | 3 | ^183^ |
| Balis (1998) | Non-compartmental pharmacokinetics (MTX p.o., plasma MTX concentrations) | 89 / 0 | 4.6 (1.1-17.3) | No effect of age on CL (mL/min/m^2^) of MTX. | 3 | ^171^ |
| ***Methotrexate (high dose)*** | | | | | | |
| Goh (1979) | Non-compartmental pharmacokinetics (plasma MTX) | 24 / 0 | NS (1.3-20) | Effect of age was not studied. | 3 | ^184^ |
| Ettinger (1982) | Non-compartmental pharmacokinetics (plasma MTX) | 16 / 0 | NS (1.75-14) | Effect of age was not studied. | 3 | ^185^ |
| Evans (1984) | Non-compartmental pharmacokinetics (plasma MTX) | 108 / NS | 4.1 (0.4-17) | No effect of age on CL (mL/min/m^2^). | 3 | ^186^ |
| Parker (1986) | Non-compartmental pharmacokinetics (plasma MTX) | 12 / 0 | 6 (2-18) | No effect of age on plasma concentrations. | 3 | ^187^ |
| Slørdal (1987) | Non-compartmental pharmacokinetics (plasma MTX) | 5 / 0 | 6.2 (2.5-12), mean (range) | Effect of age was not studied. | 3 | ^188^ |
| Borsi (1987) | Non-compartmental pharmacokinetics (plasma MTX) | 58 / 0 | 4 (1-19) | Children <4 years had a lower steady state concentration, higher V (L/m^2^) and higher CL (mL/min/m^2^) than older children. | 3 | ^189^ |
| Wolfrom (1990) | Non-compartmental pharmacokinetics (plasma MTX, 7OH-MTX) | 5 / 0 | NS (2-7) | Effect of age was not studied. | 3 | ^190^ |
| Borsi (1990) | Non-compartmental pharmacokinetics (plasma MTX, 7OH-MTX) | 58 / 0 | 4 (1-19) | A correlation between metabolic index and age: Younger patients had higher metabolite concentrations. | 3 | ^191^ |
| McLeod (1992) | Two compartment PK model (plasma MTX) | 112 / 4 | NS (0.26-19) | CL (mL/min/m^2^) tended to be lower in infants compared to older children (not significant). | 2 | ^81^ |
| Najjar (1993) | Non-compartmental pharmacokinetics (plasma MTX) | 10 / 0 | 4.8 (3-12) | No effect of age on CL (mL/min/m^2^). | 3 | ^192^ |
| Murry (1995) | Two compartment PK model (plasma MTX) | 18 / NS | 7.8 (0.2-17.2) | Effect of age was not studied. | 3 | ^193^ |
| Donelli (1995) | Two compartment PK model (plasma MTX) | 122 / 7 | NS (0.25-15) | Patients <10 years had a higher CL (L/h/kg and L/h/m^2^). A faster elimination in infants was observed. | 2 | ^194^ |
| Seidel (1997) | Non-compartmental pharmacokinetics (plasma MTX) | 42 / NS | NS (0.8-13.1) | Effect of age was not studied. | 3 | ^195^ |
| Rask (1998) | Non-compartmental pharmacokinetics (plasma MTX, 7OH-MTX) | 13 / 0 | 6.7 (3.3-12.9) | No effect of age on AUC. | 3 | ^196^ |
| Odoul (1999) | Two compartment PK model (plasma MTX) | 23 / 4 (Fig. 4) | 6 (0.75-15), mean (range) | There was a correlation between V (L) and age and between CL (L/h) and BW. | 2 | ^197^ |
| Wall (2000) | Two compartment popPK model (plasma MTX) | 24 / 0 | 12.5 (1.5-22) | Patients <6 years had a higher CL (mL/min/m^2^). | 2 | ^198^ |
| Seidel (2000) | Non-compartmental pharmacokinetics (plasma MTX) | 42 / 1 | NS (0.83-13) | Variability of plasma concentrations was higher in younger patients. | 3 | ^199^ |
| Crews (2004) | Two compartment PK model (plasma MTX) | 140 / 0 | 14.5 (3.2-24.1) | Age did not influence Cmax, AUC or CL (mL/min/m^2^). | 2 | ^200^ |
| Aumente (2006) | Two compartment popPK model (plasma MTX)  Age and BW were included as covariate on CL and V1. | 49 / 2 | 5.0 (0.5-17) | Age was incorporated as categorical covariate on CL and V1 (≤10 yrs and >10 yrs). | 1 | ^201^ |
| Thompson (2007) | Non-compartmental pharmacokinetics (plasma MTX) | 61 / 61 | 0.63 (0.17-1), mean (range) | CL (mL/min/m^2^) in infants of 0-6 months was lower than in infants of 7-12 months. Older infants showed similar CL values compared to older children. | 3 | ^202^ |
| Piard (2007) | Two compartment popPK model (plasma MTX)  BW was included as covariate on V1. | 79 / 0 | 6.9 (2-16), mean (range) | Significant correlation between V1 and BW. | 2 | ^203^ |
| Lönnerholm (2009) | Non-compartmental pharmacokinetics (plasma MTX) | 103 / 85 | 0.69 (0.16-1.16) | CL increases with age | 3 | ^204^ |
| Chládková (2010) | Two compartment popPK model (plasma MTX, 7OH-MTX) | 10 / 0 | 8.5 (2.9-16), mean (range) | Effect of age was not studied. | 2 | ^205^ |
| Martelli (2011) | Two compartment popPK model (plasma MTX) | 69 / 0 | 6.7 (1-15) | Effect of age was not described. | 2 | ^206^ |
| Jönsson (2011) | Two compartment popPK model (plasma MTX)  BW was included as covariate on the PK parameters. | 340 / NS | 5.0 (0.44-17.83) | After including BW, no effect of age on the parameters was observed. | 1 | ^207^ |
| Rühs (2012) | Two compartment popPK model (plasma MTX)  Age- and gender-normalized creatinine clearance was included as covariate on CL. | 494 / 0 | 5.42 (1.03-18.85) | Effect of age was not studied. | 2 | ^208^ |
| Csordas (2013) | Non-compartmental pharmacokinetics (plasma MTX, 7OH-MTX) | 153 / 0 | 6.4 (1.0-17.9), mean (range) | Children >14 years had a significantly higher MTX concentration at 48 h than children <6 years of age | 3 | ^209^ |
| Wright (2015) | Two compartment popPK model (plasma MTX)  Age was included as covariate on CL and V1. | 75 / 22 (Fig. 2) | 1.6 (0.02-3.5) | CL and V1 increased with age. | 1 | ^210^ |
| Lucchesi (2016) | Non-compartmental pharmacokinetics (plasma MTX) | 8 / 8 | 0.4 (0-0.75) | No significant correlation between age and CL (L/h/m^2^) | 3 | ^211^ |
| Beechinor (2019) | Two compartment popPK model (plasma MTX)  Allometric scaling for BW. | 71 / 71 | 0.71 (0.24-1.08) | No effect of age on the PK parameters. | 1 | ^212^ |
| Medellin-Garibay (2019) | Two compartment popPK model (plasma MTX)  BSA was included as covariate on CL. | 50 / 0 | 5 (1-15) | No effect of age on the PK parameters. | 2 | ^213^ |
| Hui (2019) | Two compartment popPK model (plasma MTX)  For ALL patients: BW and creatinine clearance were included as covariate on CL. Age was included as covariate on Q.  For osteosarcoma patients: Height was included as covariate on CL and V1. Dose/BSA and creatinine clearance were included as covariate on CL. BW was included as covariate on Q. Age was included as covariate on V2. | 52 / 0 | NS (1.3-19) | No effect of age after including covariates. | 2 | ^214^ |
| Kawakatsu (2019) | Two compartment popPK model (plasma MTX)  Allometric scaling for BW.  eGFR was included as covariate on CL. Age and ALT were included as covariate on V1. ALT was included as covariate on V2. | 320 / NS | 16.4 (0.6-78.9) | Age was included as covariate on V1, indicating a lower V1, corrected for BW, in older patients. | 1 | ^215^ |
| Panetta (2020) | Two compartment popPK model (plasma MTX)  PK parameters were normalized to BSA.  eGFR and treatment with dexamethasone or vancomycin were included as covariates on CL. | 178 / 55 | 1.8 (0.02-4.7) | CL in infants was lower than in older children. | 1 | ^216^ |
| Shi (2020) | One compartment popPK model (plasma MTX)  BW, creatinine clearance and treatment with dexamethasone were included as covariates on CL. | 105 / NS | 3 (0-15), mean (range) | No effect of age after including covariates. | 1 | ^217^ |
| Schulte (2021) | Two compartment popPK model (plasma MTX)  Allometric scaling for BW.  SLCO1B1 polymorphism was included as covariate on CL. | 106 / 2 | 10.1 (0.6-27.6) | No effect of age after including covariates. | 1 | ^218^ |
| Gao (2021) | Three compartment popPK model  Allometric scaling for BW.  Serum creatinine was included as covariate on CL. | 311 / NS | 5.0 (0.75-15.2) | No effect of age or maturation after including covariates. | 1 | ^219^ |
| ***Mitoxantrone*** | | | | | | |
| Lacayo (2002) | Three compartment popPK model  Cyclosporine treatment was included as covariate on CL. | 12 / NS | NS (0.7-17) | Effect of age was not studied. | 2 | ^113^ |
| Balis (2017) | Mechanism-based development of dose bands based on BSA intervals | NA | NA | NA | 5 | ^5^ |
| ***Pegaspargase*** | | | | | | |
| Muller (2000) | Comparison of asparaginase activity after single i.v. dose using a popPK model (not published). | 70 / NS | 6 (0.4-17) | Effect of age was not studied. | 3 | ^220^ |
| Avramis (2002) | One compartment popPK model (pegaspargase i.m.) | 59 / 0 | NS (1-9) | There was no correlation between V, the half-lives of absorption or elimination and BSA or age. | 2 | ^221^ |
| Vieira Pinheiro (2002) | Non-compartmental pharmacokinetics (pegaspargase i.v.) | 271 / 1 | NS (0.9-19) | No clear effect of age on PK was described. | 3 | ^222^ |
| Vieira Pinheiro (2006) | Non-compartmental pharmacokinetics (pegaspargase i.v.) | 70 / 0 | 4.6 (1.7-14) | Effect of age was not studied. | 3 | ^223^ |
| Appel (2008) | Non-compartmental pharmacokinetics (pegaspargase i.v.) | 57 / 0 | 4.9 (1.4-15.1) | No correlation with peak levels of pegaspargase and age was observed. | 3 | ^224^ |
| Hempel (2010) | One compartment popPK model (pegaspargase i.v.)  BSA was included as covariate on CL and V.  CL increased with time. | 168 / 2 (Fig. 4) | 6.7 (0-20) | After including BSA as covariate on CL and V, the influence of age was not significant. However, a trend towards higher V in the older patients was found (not significant). | 1 | ^225^ |
| Tram Henriksen (2017) | Non-compartmental pharmacokinetics (pegaspargase i.m.) | 97 / 0 | 4 (1-17) | Effect of age was not studied. | 3 | ^226^ |
| Würthwein (2017) | CL increasing with time, described by using a transit compartment model (pegaspargase i.v.) | 1342 / 0 | 5.2 (1.0-17.9) | Effect of age was not studied. | 3 | ^227^ |
| Albertsen (2019) | One compartment popPK model (pegaspargase i.m.) | 11 / 4 | At diagnosis: 0.72 (0.29-0.94) | The half-life and V (L/m^2^) being the same in infants and children, the higher mean asparaginase activity value reported in this study reflects a 200 and 250% higher dose. CL seems to be the same in these age groups. | 1 | ^228^ |
| Schore (2019) | Non-compartmental pharmacokinetics (pegaspargase i.v.) | 48 / 0 | 10.7 (1.08-23.49) | Effect of age was not studied. | 3 | ^229^ |
| Kloos (2020) | One compartment popPK model (pegaspargase i.v.)  Significant covariates for CL were: BSA, infection and treatment phase | 120 / 0 | NS (3.3-12.5) | No effect of age on the PK parameters. | 2 | ^230^ |
| ***Prednisolone*** | | | | | | |
| Choonara (1989) | Non-compartmental pharmacokinetics | 6 / 0 | 4.75 (2.8-12.4) | No effect of age on PK in these 6 patients. But values here for CL (mL/min/kg) are slightly higher than those reported in studies with older children. | 3 | ^231^ |
| Hill (1990) | Non-compartmental pharmacokinetics | 43 / 0 | 10 (2-50), mean (range) | Significant negative correlation between BSA-normalized CL and age. Children <12 years old had higher CL than older children and adults. | 3 | ^232^ |
| Petersen (2003) | Two compartment popPK model  BSA was included as covariate on CL. BW was included as covariate on V1 and V2. | 23 / 0 | 5.4 (2.4-15.2) | No additional effect of age on PK parameters that was not explained by BSA or BW. | 2 | ^233^ |
| Sassen (2021) | One compartment popPK model  Allometric scaling for BW.  Plasma protein binding of prednisolone and the ratio of  prednisolone/prednisone was included as covariate. | 124 / 1 (Fig. 3) | 6.2 (0.4–17.7) | The plasma protein binding of prednisolone to corticosteroid-binding globulin was associated with patient age. The estimated corticosteroid-binding globulin concentration decreased with age. No correlation between CL of unbound prednisolone (corrected for BSA) and age was observed. | 1 | ^234^ |
| ***Temozolomide*** | | | | | | |
| Panetta (2003) | One compartment popPK model  BSA and age were included as covariates on CL and V. | 39 / NS | 7.1 (0.7-21.9) | Impact of increasing age and BSA on CL (L/h) and V (L). | 1 | ^235^ |
| Riccardi (2003) | Non-compartmental pharmacokinetics | 22 (children) / 0  8 (adults) | 40 (3-16) /  30 (19-54), mean (range) | No effect of age on CL. BSA-normalized CL values comparable between children and adults. | 3 | ^236^ |
| Wagner (2004) | One compartment popPK model | 12 / 0 | 12.5 (1-23) | Effect of age was not studied. | 2 | ^136^ |
| Broniscer (2005) | One compartment popPK model | 33 / 0 | 6.4 (3.1-15) | Effect of age was not studied. | 2 | ^237^ |
| Kirstein (2005) | One compartment popPK model | 38 / NS | NS | Effect of age was not studied. | 2 | ^238^ |
| Horton (2007) | Non-compartmental pharmacokinetics and compartmental PK model | 16 / 0 | 11 (1-19) | Effect of age was not studied. | 3 | ^239^ |
| Broniscer (2007) | One compartment popPK model | 44 / NS (Stratum 1)  26 / 0 (Stratum 2) | 8.6 (0.4-20.2) S1  11.3 (2.4-18.6) S2 | Effect of age was not studied. | 2 | ^240^ |
| Meany (2009) | Non-compartmental pharmacokinetics | 21 / 0 | 11.4 (3.0-21.3) | No effect of age on BSA-normalized CL | 3 | ^241^ |
| Rubie (2010) | One compartment popPK model | 16 / 0 | 8.5 (3-19) | Effect of age was not studied. | 2 | ^242^ |
| ***Thioguanine*** | | | | | | |
| Erb (1998) | Non-compartmental pharmacokinetics (RBC 6-TGN) | 22 / 0 | NS (1-18) | Effect of age was not studied. | 3 | ^172^ |
| Lancaster (2001) | Non-compartmental pharmacokinetics (plasma and RBC 6-TGN) | 11 / 0 | 4 (1.5-7) | Effect of age was not studied. | 3 | ^243^ |
| Lowe (2001) | Non-compartmental pharmacokinetics (plasma and RBC 6-TGN) | 35 / 0 | 3 (1-9) | No effect of age on AUC of plasma 6-TGN was found. | 3 | ^244^ |
| Palle (2009) | Non-compartmental pharmacokinetics (RBC 6-TGN) | 46 / 2 | 11.0 (0.5-17.7) | No effect of age on RBC 6-TGN concentrations have been found. | 3 | ^245^ |
| ***Topotecan*** | | | | | | |
| Blaney (1993) | Non-compartmental pharmacokinetics | 14 / 0 | 16 (1-23) | Effect of age was not studied. | 3 | ^246^ |
| Pratt (1994) | Two compartment PK model | 14 / 0 | 10 (2-20) | Effect of age was not studied. | 3 | ^247^ |
| Stewart (1994) | Two compartment PK model | 20 / 0 | 8 (3.5-18.0) | No correlation between age and PK parameters. | 3 | ^248^ |
| Baker (1995) | Three compartment PK model including CSF | 17 / 0 | 12 (1-16) | Effect of age was not studied. | 3 | ^249^ |
| Furman (1996) | Two compartment popPK model | 18 / 0 | 10.8 (1.3-20.1) | Effect of age was not studied. | 2 | ^250^ |
| Tubergen (1996) | Two compartment PK model | 36 / 0 | 11.3 (3.1-20.6) | No correlation between age and PK parameters. | 3 | ^251^ |
| Frangoul (1999) | Non-compartmental pharmacokinetics | 15 / 0 | 9 (2-18) | Effect of age was not studied. | 3 | ^252^ |
| Athale (2002) | Two compartment PK model | 16 / 0 | 13 (1.5-21) | Effect of age was not studied. | 3 | ^253^ |
| Furman (2002) | Two compartment PK model | 33 / 0 | 9.2 (1.9-20.4) | A correlation between age and CL (L/h/m^2^) was found. | 3 | ^254^ |
| Santana (2003) | Two compartment PK model | 15 / 0 | 12.8 (2.1-19) | Effect of age was not studied. | 3 | ^255^ |
| Daw (2004) | Two compartment popPK model (after oral administration) | 20 / 0 | 10.6 (3.8-19.8) | Effect of age was not studied. | 2 | ^256^ |
| Stewart (2004) | Two (three including CSF) compartment PK model | 36 / 0 | 7.3 (3.2-16.9) | Effect of age was not studied. | 3 | ^257^ |
| Santana (2005) | Two compartment PK model | 30 / NS | 3.1 (<0.08-16.9) | Effect of age was not studied. | 3 | ^258^ |
| Freeman (2006) | Two (three including CSF) compartment PK model | 6 / 0 | 4.9 (3.2-8.4) | Effect of age was not studied. | 3 | ^259^ |
| Schaiquevich (2007) | Two compartment popPK model  Significant covariates for CL were age, renal function, and administration of phenytoin or dexamethasone.  Significant covariates for V1 were age and administration of phenytoin. | 162 / 16 or 17 (Fig. 2) | 8.0 (0.04-22) | Age evaluated as a linear continuous variable was not a significant covariate.  Age evaluated as a categorical variable (i.e., age <0.5 years and age >0.5 years) was a significant covariate for BSA-normalized CL and V1. | 1 | ^260^ |
| Hijiya (2008) | Two compartment PK model | 23 / NS | 12.7 (0.6-21.1) | Effect of age was not studied. | 3 | ^261^ |
| Rubie (2010) | Two compartment popPK model | 16 / 0 | 8.5 (3-19) | Effect of age was not studied. | 2 | ^242^ |
| Roberts (2016) | One compartment popPK model (after oral administration)  BSA was included as covariate on CL and V. ABCG2 polymorphism was included as covariate on the absorption rate constant. | 61 / NS  (20 <2 years) | 2.37 (0.48-4.59) | No effect of age on the PK parameters. | 1 | ^262^ |
| Balis (2017) | Mechanism-based development of dose bands based on BSA intervals | NA | NA | NA | 5 | ^5^ |
| ***Vincristine*** | | | | | | |
| Crom (1994) | Two compartment PK model | 54 / 2 | 4.3 (0.2-18) | No correlation between CL (mL/min/m^2^) and age. A significant correlation between CL (mL/min/kg) and age.  CL in children in higher than in adults, but CL in two infants was lower. | 2 | ^263^ |
| De Graaf (1995) | Two compartment PK model | 17 / 0 | 3.8 (1.3-12.4) | CL (mL/min/m^2^) in children appeared to be more than twice as large as in adults and t_1/2_ in adults was much longer than in children. | 3 | ^264^ |
| Gidding (1999) | Two compartment PK model | 32 / NS | 4.6 (0-16) | CL (mL/min/m^2^) in infants under 1 year is lower than in older children. | 2 | ^265^ |
| Groninger (2002) | Two compartment PK model | 70 / 0 | NS (1-16) | No effect of age on CL (mL/min/m^2^). | 3 | ^266^ |
| Frost (2003) | Two compartment PK model | 98 / 0 | 4.5 (1.3-17.3) | No effect of age on the PK parameters. | 3 | ^267^ |
| Plasschaert (2004) | Two compartment PK model | 52 / 0 | NS (1-16) | No effect of age on CL (mL/min/m^2^). | 3 | ^268^ |
| Groninger (2005) | Two compartment PK model | 54 / 0 | NS (1-16) | Effect of age was not studied. | 3 | ^269^ |
| Lönnerholm (2008) | Two compartment PK model (with data of Frost 2003) | 86 / 0 | NS (1.2-17.4) | No effect of age on the PK parameters. | 3 | ^270^ |
| Guilhaumou (2011) | Two compartment popPK model | 26 / 0 | NS (2.0-16.0) | No effect of age on the PK parameters. | 2 | ^271^ |
| Moore (2011) | One compartment popPK model  Allometric scaling for BW. | 50 / 0 | 6.5 (1.0-16.25) | No effect of age on the PK parameters. | 2 | ^272^ |
| Balis (2017) | Mechanism-based development of dose bands based on BSA intervals | NA | NA | NA | 5 | ^5^ |
| Lee (2019) | Physiological-based PK model  Intracellular binding to β-tubulin was included as covariate. | 25 / NS | NS (0.4-9) | Simulating a higher hypothetical (4.9-fold) pediatric expression of β-tubulin relative to adult improved predictions PK. | 5 | ^273^ |
| Van de Velde (2020) | Two compartment popPK model  PK parameters were normalized to BSA. Duration of infusion was included as covariate on Q and V2. | 35 / 0 | 10.06 (NS) | Effect of age was not described. | 2 | ^274^ |
| Skolnik (2021) | Non-compartmental pharmacokinetics | 132 / 9 | NS (0.21-16.8) | Age had a minimal effect on variability of PK. Compared to children <1 year, the BSA-adjusted dose was 44-79% higher older children. Consistent with the differences in dose, the median AUC was lowest for children <1 year and highest in older children. CL did not appear to be related to age. | 3 | ^6^ |
| Barnett (2021) | Two compartment popPK model  Allometric scaling for BW.  Age was included as covariate on V2 | 57 / 21 | 5.6 (0.04-17.2) | No significant difference in BSA-normalized CL between infants and older children. There was a trend towards lower CL in neonates (0-4 weeks) as compared to infants (1-12 months).  Doses of <0.05mg/kg result in significantly lower AUC values than observed in neonates and infants receiving doses of ≥0.05mg/kg, and in older children receiving a dose of 1.5mg/m^2^. | 1 | ^275^ |

AUC Area under the curve; BSA Body surface area; BW Body weight; CL Clearance; Cmax Maximum plasma concentration; eGFR estimated glomerular filtration rate; NS Not specified; PK Pharmacokinetic(s); popPK Population pharmacokinetic(s); Q Intercompartmental clearance; RBC Red blood cell count; t_1/2_ elimination half-life; V Volume of distribution.

**References**

1. Veal GJ, Cole M, Errington J, et al. Pharmacokinetics of Dactinomycin in a Pediatric Patient Population: a United Kingdom Children’s Cancer Study Group Study. *Clin Cancer Res*. 2005;11(16):5893-5899. doi:10.1158/1078-0432.CCR-04-2546

2. Mondick JT, Gibiansky L, Gastonguay MR, et al. Population pharmacokinetic investigation of actinomycin-D in children and young adults. *J Clin Pharmacol*. 2008;48(1):35-42. doi:10.1177/0091270007310383

3. Edwards AYZ, Skolnik JM, Dombrowsky E, Patel D, Barrett JS. Modeling and simulation approaches to evaluate pharmacokinetic sampling contamination from central venous catheters in pediatric pharmacokinetic studies of actinomycin-D: a report from the children’s oncology group. *Cancer Chemother Pharmacol*. 2012;70(1):83-94. doi:10.1007/s00280-012-1878-y

4. Hill CR, Cole M, Errington J, Malik G, Boddy A V., Veal GJ. Characterisation of the Clinical Pharmacokinetics of Actinomycin D and the Influence of ABCB1 Pharmacogenetic Variation on Actinomycin D Disposition in Children with Cancer. *Clin Pharmacokinet*. 2014;53(8):741-751. doi:10.1007/s40262-014-0153-2

5. Balis FM, Womer RB, Berg S, Winick N, Adamson PC, Fox E. Dosing anticancer drugs in infants: Current approach and recommendations from the Children’s Oncology Group’s Chemotherapy Standardization Task Force. *Pediatr Blood Cancer*. 2017;64(11):e26636. doi:10.1002/pbc.26636

6. Skolnik J, Hall D, Barkauskas DA, et al. Toxicity and pharmacokinetics of actinomycin-D and vincristine in children and adolescents: Children’s Oncology Group Study ADVL06B1. *Cancer Chemother Pharmacol*. Published online 2021. doi:10.1007/s00280-021-04295-1

7. von Stackelberg A, Locatelli F, Zugmaier G, et al. Phase I/Phase II Study of Blinatumomab in Pediatric Patients With Relapsed/Refractory Acute Lymphoblastic Leukemia. *J Clin Oncol*. 2016;34(36):4381-4389. doi:10.1200/JCO.2016.67.3301

8. Clements JD, Zhu M, Kuchimanchi M, Terminello B, Doshi S. Population Pharmacokinetics of Blinatumomab in Pediatric and Adult Patients with Hematological Malignancies. *Clin Pharmacokinet*. 2020;59(4):463-474. doi:10.1007/s40262-019-00823-8

9. Dalle JH, Wall D, Theoret Y, et al. Intravenous busulfan for allogeneic hematopoietic stem cell transplantation in infants: Clinical and pharmacokinetic results. *Bone Marrow Transplant*. 2003;32(7):647-651. doi:10.1038/sj.bmt.1704209

10. Nguyen L, Fuller D, Lennon S, Leger F, Puozzo C. I.V. busulfan in pediatrics: A novel dosing to improve safety/efficacy for hematopoietic progenitor cell transplantation recipients. *Bone Marrow Transplant*. 2004;33(10):979-987. doi:10.1038/sj.bmt.1704446

11. Tran H, Petropoulos D, Worth L, et al. Pharmacokinetics and individualized dose adjustment of intravenous busulfan in children with advanced hematologic malignancies undergoing allogeneic stem cell transplantation. *Biol Blood Marrow Transplant*. 2004;10(11):805-812. doi:10.1016/j.bbmt.2004.07.010

12. Oechtering D, Schiltmeyer B, Hempel G, et al. Toxicity and pharmacokinetics of i.v. busulfan in children before stem cell transplantation. *Anticancer Drugs*. 2005;16(3):337-344. doi:10.1097/00001813-200503000-00014

13. Kletzel M, Jacobsohn D, Duerst R. Pharmacokinetics of a test dose of Intravenous busulfan guide dose modifications to achieve an optimal area under the curve of a single daily dose of intravenous busulfan in children undergoing a reduced-intensity conditioning regimen with hematopoietic s. *Biol Blood Marrow Transplant*. 2006;12(4):472-479. doi:10.1016/j.bbmt.2005.12.028

14. Zwaveling J, Den Hartigh J, Lankester AC, Guchelaar HJ, Egeler RM, Bredius RG. Erratum: Once-daily intravenous busulfan in children prior to stem cell transplantation: Study of pharmacokinetics and early clinical outcomes (Anti-Cancer Drugs (2006) 17 (1099-1105)). *Anticancer Drugs*. 2006;17(10):1235. doi:10.1097/01.cad.0000236320.88951.e2

15. Booth BP, Rahman A, Dagher R, et al. Population pharmacokinetic-based dosing of intravenous busulfan in pediatric patients. *J Clin Pharmacol*. 2007;47(1):101-111. doi:10.1177/0091270006295789

16. Schechter T, Finkelstein Y, Doyle J, et al. Pharmacokinetic Disposition and Clinical Outcomes in Infants and Children Receiving Intravenous Busulfan for Allogeneic Hematopoietic Stem Cell Transplantation. *Biol Blood Marrow Transplant*. 2007;13(3):307-314. doi:10.1016/j.bbmt.2006.10.026

17. Nath CE, Earl JW, Pati N, Stephen K, Shaw PJ. Variability in the pharmacokinetics of intravenous busulphan given as a single daily dose to paediatric blood or marrow transplant recipients. *Br J Clin Pharmacol*. 2008;66(1):50-59. doi:10.1111/j.1365-2125.2008.03166.x

18. Kim AH, Tse JC, Ikeda A, Moore TB. Evaluating pharmacokinetics and pharmacodynamics of intravenous busulfan in pediatric patients receiving bone marrow transplantation. *Pediatr Transplant*. 2009;13(8):971-976. doi:10.1111/j.1399-3046.2008.01098.x

19. Wall DA, Chan KW, Nieder ML, et al. Safety, efficacy, and pharmacokinetics of intravenous busulfan in children undergoing allogeneic hematopoietic stem cell transplantation. *Pediatr Blood Cancer*. Published online 2009. doi:10.1002/pbc.22227

20. Trame MN, Bergstrand M, Karlsson MO, Boos J, Hempel G. Population pharmacokinetics of busulfan in children: Increased evidence for body surface area and allometric body weight dosing of busulfan in children. *Clin Cancer Res*. 2011;17(21):6867-6877. doi:10.1158/1078-0432.CCR-11-0074

21. Bartelink IH, Boelens JJ, Bredius RGM, et al. Body weight-dependent pharmacokinetics of busulfan in paediatric haematopoietic stem cell transplantation patients: Towards individualized dosing. *Clin Pharmacokinet*. 2012;51(5):331-345. doi:10.2165/11598180-000000000-00000

22. Bartelink IH, Van Kesteren C, Boelens JJ, et al. Predictive performance of a busulfan pharmacokinetic model in children and young adults. *Ther Drug Monit*. 2012;34(5):574-583. doi:10.1097/FTD.0b013e31826051bb

23. Michel G, Valteau-Couanet D, Gentet J-C, et al. Weight-based strategy of dose administration in children using intravenous busulfan: Clinical and pharmacokinetic results. *Pediatr Blood Cancer*. 2012;58(1):90-97. doi:10.1002/pbc.22959

24. Paci A, Vassal G, Moshous D, et al. Pharmacokinetic behavior and appraisal of intravenous busulfan dosing in infants and older children: The results of a population pharmacokinetic study from a large pediatric cohort undergoing hematopoietic stem-cell transplantation. *Ther Drug Monit*. 2012;34(2):198-208. doi:10.1097/FTD.0b013e31824c2f60

25. Veal GJ, Nguyen L, Paci A, et al. Busulfan pharmacokinetics following intravenous and oral dosing regimens in children receiving high-dose myeloablative chemotherapy for high-risk neuroblastoma as part of the HR-NBL-1/SIOPEN trial. *Eur J Cancer*. 2012;48(16):3063-3072. doi:10.1016/j.ejca.2012.05.020

26. Le Gall JB, Milone MC, Waxman IM, et al. The pharmacokinetics and safety of twice daily i.v. BU during conditioning in pediatric allo-SCT recipients. *Bone Marrow Transplant*. 2013;48(1):19-25. doi:10.1038/bmt.2012.105

27. McCune JS, Baker KS, Blough DK, et al. Variation in prescribing patterns and therapeutic drug monitoring of intravenous busulfan in pediatric hematopoietic cell transplant recipients. *J Clin Pharmacol*. 2013;53(3):264-275. doi:10.1177/0091270012447196

28. Savic RM, Cowan MJ, Dvorak CC, et al. Effect of weight and maturation on busulfan clearance in infants and small children undergoing hematopoietic cell transplantation. *Biol Blood Marrow Transplant*. 2013;19(11):1608-1614. doi:10.1016/j.bbmt.2013.08.014

29. Diestelhorst C, Boos J, McCune JS, Hempel G. Population pharmacokinetics of intravenous busulfan in children: Revised body weight-dependent NONMEM® model to optimize dosing. *Eur J Clin Pharmacol*. 2014;70(7):839-847. doi:10.1007/s00228-014-1692-z

30. McCune JS, Bemer MJ, Barrett JS, Baker KS, Gamis AS, Holford NHG. Busulfan in infant to adult hematopoietic cell transplant recipients: A population pharmacokinetic model for initial and bayesian dose personalization. *Clin Cancer Res*. 2014;20(3):754-763. doi:10.1158/1078-0432.CCR-13-1960

31. Okamoto Y, Nagatoshi Y, Kosaka Y, et al. Prospective pharmacokinetic study of intravenous busulfan in hematopoietic stem cell transplantation in 25 children. *Pediatr Transplant*. 2014;18(3):294-301. doi:10.1111/petr.12236

32. Long-Boyle JR, Savic R, Yan S, et al. Population pharmacokinetics of busulfan in pediatric and young adult patients undergoing hematopoietic cell transplant: A model-based dosing algorithm for personalized therapy and implementation into routine clinical use. *Ther Drug Monit*. 2015;37(2):236-245. doi:10.1097/FTD.0000000000000131

33. Neely M, Philippe M, Rushing T, et al. Accurately Achieving Target Busulfan Exposure in Children and Adolescents With Very Limited Sampling and the BestDose Software. *Ther Drug Monit*. 2016;38(3):332-342. doi:10.1097/FTD.0000000000000276

34. Nava T, Kassir N, Rezgui MA, et al. Incorporation of GSTA1 genetic variations into a population pharmacokinetic model for IV busulfan in paediatric hematopoietic stem cell transplantation. *Br J Clin Pharmacol*. 2018;84(7):1494-1504. doi:10.1111/bcp.13566

35. Alsultan A, Albassam AA, Alturki A, et al. Population pharmacokinetics of busulfan in Saudi pediatric patients undergoing hematopoietic stem cell transplantation. *Int J Clin Pharm*. 2020;42(2):703-712. doi:10.1007/s11096-020-00989-3

36. Marsit H, Philippe M, Neely M, et al. Intra-individual Pharmacokinetic Variability of Intravenous Busulfan in Hematopoietic Stem Cell-Transplanted Children. *Clin Pharmacokinet*. 2020;59(8):1049-1061. doi:10.1007/s40262-020-00877-z

37. Poinsignon V, Faivre L, Nguyen L, et al. New dosing nomogram and population pharmacokinetic model for young and very young children receiving busulfan for hematopoietic stem cell transplantation conditioning. *Pediatr Blood Cancer*. 2020;67(10):1-10. doi:10.1002/pbc.28603

38. Yuan J, Sun N, Feng X, et al. Optimization of busulfan dosing regimen in pediatric patients using a population pharmacokinetic model incorporating gst mutations. *Pharmgenomics Pers Med*. 2021;14:253-268. doi:10.2147/PGPM.S289834

39. Neroutsos E, Athanasiadou I, Paisiou A, et al. Dose individualization of intravenous busulfan in pediatric patients undergoing bone marrow transplantation: impact and in vitro evaluation of infusion lag-time. *J Pharm Pharmacol*. 2021;XX(July):1-11. doi:10.1093/jpp/rgab087

40. Ben Hassine K, Nava T, Théoret Y, et al. Precision dosing of intravenous busulfan in pediatric hematopoietic stem cell transplantation: Results from a multicenter population pharmacokinetic study. *CPT Pharmacometrics Syst Pharmacol*. Published online 2021. doi:10.1002/psp4.12683

41. Riccardi R, Riccardi A, Di Rocco C, et al. Cerebrospinal fluid pharmacokinetics of carboplatin in children with brain tumors. *Cancer Chemother Pharmacol*. 1992;30(1):21-24. doi:10.1007/BF00686480

42. Madden T, Sunderland M, Santana VM, Rodman JH. The pharmacokinetics of high-dose carboplatin in pediatric patients with cancer. *Clin Pharmacol Ther*. 1992;51(6):701-707. doi:10.1038/clpt.1992.82

43. Newell DR, Pearson a. D, Balmanno K, et al. Carboplatin pharmacokinetics in children: the development of a pediatric dosing formula. The United Kingdom Children’s Cancer Study Group. *J Clin Oncol*. 1993;11(12):2314-2323. doi:10.1200/JCO.1993.11.12.2314

44. Riccardi R, Riccardi A, Lasorella A, et al. Clinical pharmacokinetics of carboplatin in children. *Cancer Chemother Pharmacol*. 1994;33(6):477-483. doi:10.1007/BF00686504

45. Peng B, Boddy A V, Cole M, et al. Comparison of methods for the estimation of carboplatin pharmacokinetics in paediatric cancer patients. *Eur J Cancer*. 1995;31A(11):1804-1810. doi:10.1016/0959-8049(95)00382-s

46. Chatelut E, Boddy A V., Peng B, et al. Population pharmacokinetics of carboplatin in children. *Clin Pharmacol Ther*. 1996;59(4):436-443. doi:10.1016/S0009-9236(96)90113-7

47. Tonda ME, Heideman RL, Petros WP, Friedman HS, Murry DJ, Rodman JH. Carboplatin pharmacokinetics in young children with brain tumors. *Cancer Chemother Pharmacol*. 1996;38(5):395-400. doi:10.1007/s002800050502

48. Doz F, Urien S, Chatelut E, et al. A limited-sampling method for evaluation of the area under the curve of ultrafilterable carboplatin in children. *Cancer Chemother Pharmacol*. 1998;42(3):250-254. doi:10.1007/s002800050813

49. Thomas H, Boddy A V., English MW, et al. Prospective Validation of Renal Function–Based Carboplatin Dosing in Children With Cancer: A United Kingdom Children’s Cancer Study Group Trial. *J Clin Oncol*. 2000;18(21):3614-3621. doi:10.1200/JCO.2000.18.21.3614

50. Patoux A, Bleyzac N, Boddy A V, et al. Comparison of nonlinear mixed-effect and non-parametric expectation maximisation modelling for Bayesian estimation of carboplatin clearance in children. *Eur J Clin Pharmacol*. 2001;57(4):297-303. doi:10.1007/s002280100306

51. Rubie H, Doz F, Vassal G, et al. Individual dosing of carboplatin based on drug monitoring in children receiving high-dose chemotherapy. *Eur J Cancer*. 2003;39(10):1433-1438. doi:10.1016/S0959-8049(03)00314-9

52. Kangarloo SB, Gangopadhyay SB, Syme RM, Wolff JEA, Glück S. Influence of mesna on the pharmacokinetics of cisplatin and carboplatin in pediatric cancer patients. *Med Oncol*. 2004;21(1):9-20. doi:10.1385/MO:21:1:09

53. Veal GJ, Errington J, Tilby MJ, et al. Adaptive dosing and platinum-DNA adduct formation in children receiving high-dose carboplatin for the treatment of solid tumours. *Br J Cancer*. 2007;96(5):725-731. doi:10.1038/sj.bjc.6603607

54. Levy AS, Meyers PA, Wexler LH, et al. Phase 1 and pharmacokinetic study of concurrent carboplatin and irinotecan in subjects aged 1 to 21 years with refractory solid tumors. *Cancer*. 2009;115(1):207-216. doi:10.1002/cncr.23992

55. Picton S V., Keeble J, Holden V, Errington J, Boddy a. V., Veal GJ. Therapeutic monitoring of carboplatin dosing in a premature infant with retinoblastoma. *Cancer Chemother Pharmacol*. 2009;63(4):749-752. doi:10.1007/s00280-008-0787-6

56. Veal GJ, Cole M, Errington J, et al. Pharmacokinetics of carboplatin and etoposide in infant neuroblastoma patients. *Cancer Chemother Pharmacol*. 2010;65(6):1057-1066. doi:10.1007/s00280-009-1111-9

57. Veal GJ, Errington J, Hayden J, et al. Carboplatin therapeutic monitoring in preterm and full-term neonates. *Eur J Cancer*. 2015;51(14):2022-2030. doi:10.1016/j.ejca.2015.07.011

58. Veal GJ, Errington J, Sastry J, et al. Adaptive dosing of anticancer drugs in neonates: facilitating evidence-based dosing regimens. *Cancer Chemother Pharmacol*. 2016;77(4):685-692. doi:10.1007/s00280-016-2975-0

59. Duong JK, Veal GJ, Nath CE, et al. Population pharmacokinetics of carboplatin, etoposide and melphalan in children: a re-evaluation of paediatric dosing formulas for carboplatin in patients with normal or mild impairment of renal function. *Br J Clin Pharmacol*. 2019;85(1):136-146. doi:10.1111/bcp.13774

60. Hawley J, Veal GJ, Errington J, McDonald LG, Tweddle DA. The use of pharmacokinetically guided carboplatin chemotherapy in a pre-term infant with neuroblastoma-associated spinal cord compression. *Pediatr Blood Cancer*. 2019;66(9):e27825. doi:10.1002/pbc.27825

61. Hong CR, Kang HJ, Moon SJ, et al. Pharmacokinetics of high-dose carboplatin in children undergoing high-dose chemotherapy and autologous stem cell transplantation with BSA-based dosing. *Bone Marrow Transpl*. 2020;55(1):137-146. doi:10.1038/s41409-019-0655-5

62. Crom WR, Evans WE, Pratt CB, et al. Cisplatin disposition in children and adolescents with cancer. *Cancer Chemother Pharmacol*. 1981;6(1):95-99. doi:10.1007/BF00253017

63. Dominici C, Petrucci F, Caroli S, Alimonti A, Clerico A, Castello M a. A pharmacokinetic study of high-dose continuous infusion cisplatin in children with solid tumors. *J Clin Oncol*. 1989;7(1):100-107. doi:10.1200/JCO.1989.7.1.100

64. Murakami T, Inoue S, Sasaki K, Fujimoto T. Studies on age-dependent plasma platinum pharmacokinetics and ototoxicity of cisplatin. *Sel Cancer Ther*. 1990;6(3):145-151. doi:10.1089/sct.1990.6.145

65. Peng B, English MW, Boddy a. V., et al. Cisplatin pharmacokinetics in children with cancer. *Eur J Cancer*. 1997;33(11):1823-1828. doi:10.1016/s0959-8049(97)00341-9

66. Thomas F, Veal GJ, El Balkhi S, et al. Therapeutic drug monitoring and dose adaptation of cisplatin in a newborn with hepatoblastoma: a case report. *Cancer Chemother Pharmacol*. 2018;82(2):361-365. doi:10.1007/s00280-018-3625-5

67. Juma FD, Koech DK, Kasili EG, Ogada T. Pharmacokinetics of cyclophosphamide in Kenyan African children with lymphoma. *Br J Clin Pharmacol*. 1984;18(1):106-107. doi:10.1111/j.1365-2125.1984.tb05032.x

68. Tasso MJ, Boddy A V., Price L, Wyllie RA, Pearson ADJ, Idle JR. Pharmacokinetics and metabolism of cyclophosphamide in paediatric patients. *Cancer Chemother Pharmacol*. 1992;30(3):207-211. doi:10.1007/BF00686313

69. Yule SM, Boddy A V., Cole M, et al. Cyclophosphamide pharmacokinetics in children. *Br J Clin Pharmacol*. 1996;41(1):13-19. doi:10.1111/j.1365-2125.1996.tb00153.x

70. Yule SM, Foreman NK, Mitchell C, Gouldon N, May P, McDowell HP. High-dose cyclophosphamide for poor-prognosis and recurrent pediatric brain tumors: a dose-escalation study. *J Clin Oncol*. 1997;15(10):3258-3265. doi:10.1200/JCO.1997.15.10.3258

71. Yule SM, Price L, Cole M, Pearson AD, Boddy a. V. Cyclophosphamide metabolism in children following a 1-h and a 24-h infusion. *Cancer Chemother Pharmacol*. 2001;47(3):222-228. doi:10.1007/s002800000220

72. Yule SM, Price L, McMahon AD, Pearson AD, Boddy A V. Cyclophosphamide metabolism in children with non-Hodgkin’s lymphoma. *Clin Cancer Res*. 2004;10(2):455-460. doi:10.1158/1078-0432.ccr-0844-03

73. McCune JS, Salinger DH, Vicini P, Oglesby C, Blough DK, Park JR. Population pharmacokinetics of cyclophosphamide and metabolites in children with neuroblastoma: a report from the Children’s Oncology Group. *J Clin Pharmacol*. 2009;49(1):88-102. doi:10.1177/0091270008325928

74. Balasubramanian P, Desire S, Panetta JC, et al. Population pharmacokinetics of cyclophosphamide in patients with thalassemia major undergoing HSCT. *Bone Marrow Transpl*. 2012;47(9):1178-1185. doi:10.1038/bmt.2011.254

75. Navid F, Baker SD, McCarville MB, et al. Phase I and clinical pharmacology study of bevacizumab, sorafenib, and low-dose cyclophosphamide in children and young adults with refractory/recurrent solid tumors. *Clin Cancer Res*. 2013;19(1):236-246. doi:10.1158/1078-0432.CCR-12-1897

76. Veal GJ, Cole M, Chinnaswamy G, et al. Cyclophosphamide pharmacokinetics and pharmacogenetics in children with B-cell non-Hodgkin’s lymphoma. *Eur J Cancer*. 2016;55:56-64. doi:10.1016/j.ejca.2015.12.007

77. Campagne O, Zhong B, Nair S, et al. Exposure–Toxicity Association of Cyclophosphamide and Its Metabolites in Infants and Young Children with Primary Brain Tumors: Implications for Dosing. *Clin Cancer Res*. 2020;26(7):1563-1573. doi:10.1158/1078-0432.CCR-19-2685

78. Barnett S, Errington J, Sludden J, et al. Pharmacokinetics and Pharmacogenetics of Cyclophosphamide in a Neonate and Infant Childhood Cancer Patient Population. *Pharm*. 2021;14(3). doi:10.3390/ph14030272

79. Avramis VI, Biener R, Krailo M, et al. Biochemical pharmacology of high dose 1-beta-D-arabinofuranosylcytosine in childhood acute leukemia. *Cancer Res*. 1987;47(24 Pt 1):6786-6792. http://www.ncbi.nlm.nih.gov/pubmed/3479250

80. Avramis VI, Weinberg KI, Sato JK, et al. Pharmacology Studies of 1-beta-D-Arabinofuranosylcytosine in Pediatric Patients with Leukemia and Lymphoma after a Biochemically Optimal Regimen of Loading Bolus plus Continuous Infusion of the Drug. *Cancer Res*. 1989;49(1):241-247.

81. McLeod HL, Relling M V, Crom WR, et al. Disposition of antineoplastic agents in the very young child. *Br J Cancer Suppl*. 1992;18:S23-29.

82. Periclou AP, Avramis VI. NONMEM population pharmacokinetic studies of cytosine arabinoside after high-dose and after loading bolus followed by continuous infusion of the drug in pediatric patients with leukemias. *Cancer Chemother Pharmacol*. 1996;39(1-2):42-50. doi:10.1007/s002800050536

83. Avramis VI, Wiersma S, Krailo MD, et al. Pharmacokinetic and pharmacodynamic studies of fludarabine and cytosine arabinoside administered as loading boluses followed by continuous infusions after a phase I/II study in pediatric patients with relapsed leukemias. The Children’s Cancer Group. *Clin Cancer Res*. 1998;4(1):45-52. http://www.ncbi.nlm.nih.gov/pubmed/9516951

84. Evans WE, Relling M V., Rodman JH, Crom WR, Boyett JM, Pui C-H. Conventional Compared with Individualized Chemotherapy for Childhood Acute Lymphoblastic Leukemia. *N Engl J Med*. 1998;338(8):499-505. doi:10.1056/NEJM199802193380803

85. Ozkaynak MF, Avramis VI, Carcich S, Ortega J a. Pharmacology of cytarabine given as a continuous infusion followed by mitoxantrone with and without amsacrine/etoposide as reinduction chemotherapy for relapsed or refractory pediatric acute myeloid leukemia. *Med Pediatr Oncol*. 1998;31(6):475-482. doi:10.1002/(sici)1096-911x(199812)31:6<475::aid-mpo3>3.0.co;2-7

86. Hempel G, Relling M V., de Rossi G, et al. Pharmacokinetics of daunorubicin and daunorubicinol in infants with leukemia treated in the interfant 99 protocol. *Pediatr Blood Cancer*. 2010;54(3):355-360. doi:10.1002/pbc.22266

87. Thompson P, Wheeler HE, Delaney SM, et al. Pharmacokinetics and pharmacogenomics of daunorubicin in children: A report from the Children’s Oncology Group. *Cancer Chemother Pharmacol*. 2014;74(4):831-838. doi:10.1007/s00280-014-2535-4

88. Spektor Z, Jasek MC, Jasheway D, et al. Pharmacokinetics of CIPRODEX otic in pediatric and adolescent patients. *Int J Pediatr Otorhinolaryngol*. 2008;72(1):97-102. doi:10.1016/j.ijporl.2007.09.017

89. Yang L, Panetta JC, Cai X, et al. Asparaginase may influence dexamethasone pharmacokinetics in acute lymphoblastic leukemia. *J Clin Oncol*. 2008;26(12):1932-1939. doi:10.1200/JCO.2007.13.8404

90. Kawedia JD, Liu C, Pei D, et al. Dexamethasone exposure and asparaginase antibodies affect relapse risk in acute lymphoblastic leukemia. *Blood*. 2012;119(7):1658-1664. doi:10.1182/blood-2011-09-381731

91. Inaba H, Cao X, Han AQ, et al. Bone mineral density in children with acute lymphoblastic leukemia. *Cancer*. 2018;124(5):1025-1035. doi:10.1002/cncr.31184

92. Jackson RK, Liebich M, Berry P, et al. Impact of dose and duration of therapy on dexamethasone pharmacokinetics in childhood acute lymphoblastic leukaemia-a report from the UKALL 2011 trial. *Eur J Cancer*. 2019;120:75-85. doi:10.1016/j.ejca.2019.07.026

93. Uttenreuther-Fischer MM, Huang CS, Yu AL. Pharmacokinetics of human-mouse chimeric anti-GD2 mAb ch14.18 in a phase I trial in neuroblastoma patients. *Cancer Immunol Immunother*. 1995;41(6):331-338. doi:10.1007/BF01526552

94. Ladenstein R, Weixler S, Baykan B, et al. Ch14.18 antibody produced in CHO cells in relapsed or refractory Stage 4 neuroblastoma patients: a SIOPEN Phase 1 study. *MAbs*. 2013;5(5):801-809. doi:10.4161/mabs.25215

95. Desai A V, Fox E, Smith LM, Lim AP, Maris JM, Balis FM. Pharmacokinetics of the chimeric anti-GD2 antibody, ch14.18, in children with high-risk neuroblastoma. *Cancer Chemother Pharmacol*. 2014;74(5):1047-1055. doi:10.1007/s00280-014-2575-9

96. Marachelian A, Desai A, Balis F, et al. Comparative pharmacokinetics, safety, and tolerability of two sources of ch14.18 in pediatric patients with high-risk neuroblastoma following myeloablative therapy. *Cancer Chemother Pharmacol*. 2016;77(2):405-412. doi:10.1007/s00280-015-2955-9

97. Eksborg S, Söderhäll S, Frostvik-Stolt M, Lindberg A, Liliemark E. Plasma pharmacokinetics of etoposide (VP-16) after i.v. administration to children. *Anticancer Drugs*. 2000;11(4):237-241. doi:10.1097/00001813-200004000-00002

98. Frost B-M, Eksborg S, Björk O, et al. Pharmacokinetics of doxorubicin in children with acute lymphoblastic leukemia: multi-institutional collaborative study. *Med Pediatr Oncol*. 2002;38(5):329-337. doi:10.1002/mpo.10052

99. Hempel G, Flege S, Würthwein G, Boos J. Peak plasma concentrations of doxorubicin in children with acute lymphoblastic leukemia or non-Hodgkin lymphoma. *Cancer Chemother Pharmacol*. 2002;49(2):133-141. doi:10.1007/s00280-001-0392-4

100. Palle J, Frost B-M, Peterson C, et al. Doxorubicin pharmacokinetics is correlated to the effect of induction therapy in children with acute myeloid leukemia. *Anticancer Drugs*. 2006;17(4):385-392. doi:10.1097/01.cad.0000198911.98442.16

101. Thompson P a., Rosner GL, Matthay KK, et al. Impact of body composition on pharmacokinetics of doxorubicin in children: a Glaser Pediatric Research Network study. *Cancer Chemother Pharmacol*. 2009;64(2):243-251. doi:10.1007/s00280-008-0854-z

102. Kontny NE, Würthwein G, Joachim B, et al. Population pharmacokinetics of doxorubicin: Establishment of a NONMEM model for adults and children older than 3 years. *Cancer Chemother Pharmacol*. 2013;71(3):749-763. doi:10.1007/s00280-013-2069-1

103. Völler S, Boos J, Krischke M, et al. Age-Dependent Pharmacokinetics of Doxorubicin in Children with Cancer. *Clin Pharmacokinet*. 2015;54(11):1139-1149. doi:10.1007/s40262-015-0272-4

104. Krischke M, Hempel G, Völler S, et al. Pharmacokinetic and pharmacodynamic study of doxorubicin in children with cancer: results of a “European Pediatric Oncology Off-patents Medicines Consortium” trial. *Cancer Chemother Pharmacol*. 2016;78(6):1175-1184. doi:10.1007/s00280-016-3174-8

105. Kunarajah K, Hennig S, Norris RLG, et al. Population pharmacokinetic modelling of doxorubicin and doxorubicinol in children with cancer: is there a relationship with cardiac troponin profiles? *Cancer Chemother Pharmacol*. 2017;80(1):15-25. doi:10.1007/s00280-017-3309-6

106. Siebel C, Würthwein G, Lanvers-Kaminsky C, et al. Can we optimise doxorubicin treatment regimens for children with cancer? Pharmacokinetic simulations and a Delphi consensus procedure. *BMC Pharmacol Toxicol*. 2020;21(1):1-10. doi:10.1186/s40360-020-00417-2

107. Evans WE, Sinkule JA, Crom WR, Dow L, Look AT, Rivera G. Pharmacokinetics of teniposide (VM26) and etoposide (VP16-213) in children with cancer. *Cancer Chemother Pharmacol*. 1982;7(2-3):147-150. doi:10.1007/BF00254537

108. Lowis SP, Pearson ADJ, Newell DR, Cole M. Etoposide Pharmacokinetics in Children: The Development and Prospective Validation of a Dosing Equation. *Cancer Res*. 1993;53(20):4881-4889.

109. Rodman JH, Murry DJ, Madden T, Santana VM. Altered etoposide pharmacokinetics and time to engraftment in pediatric patients undergoing autologous bone marrow transplantation. *J Clin Oncol*. 1994;12(11):2390-2397. doi:10.1200/JCO.1994.12.11.2390

110. Boos J, Krümpelmann S, Schulze-Westhoff P, Euting T, Berthold F, Jürgens H. Steady-state levels and bone marrow toxicity of etoposide in children and infants: Does etoposide require age-dependent dose calculation? *J Clin Oncol*. 1995;13(12):2954-2960. doi:10.1200/JCO.1995.13.12.2954

111. Würthwein G, Krümpelmann S, Tillmann B, et al. Population pharmacokinetic approach to compare oral and i.v. administration of etoposide. *Anticancer Drugs*. 1999;10(9):807-814. doi:10.1097/00001813-199910000-00003

112. Eksborg S, Söderhäll S, Frostvik-Stolt M, Lindberg A, Liliemark E. Plasma pharmacokinetics of etoposide (VP-16) after i.v. administration to children. *Anticancer Drugs*. 2000;11(4):237-241. doi:10.1097/00001813-200004000-00002

113. Lacayo NJ, Lum BL, Becton DL, et al. Pharmacokinetic interactions of cyclosporine with etoposide and mitoxantrone in children with acute myeloid leukemia. *Leukemia*. 2002;16(5):920-927. doi:10.1038/sj.leu.2402455

114. Würthwein G, Klingebiel T, Krümpelmann S, et al. Population pharmacokinetics of high-dose etoposide in children receiving different conditioning regimens. *Anticancer Drugs*. 2002;13(1):101-110. doi:10.1097/00001813-200201000-00012

115. Kato Y, Nishimura S, Sakura N, Ueda K. Pharmacokinetics of etoposide with intravenous drug administration in children and adolescents. *Pediatr Int*. 2003;45(1):74-79. doi:10.1046/j.1442-200x.2003.01675.x

116. Palle J, Frost B-M, Britt-Marie F, et al. Etoposide pharmacokinetics in children treated for acute myeloid leukemia. *Anticancer Drugs*. 2006;17(9):1087-1094. doi:10.1097/01.cad.0000231470.54288.49

117. Urien S, Doz F, Giraud C, et al. Developmental pharmacokinetics of etoposide in 67 children: lack of dexamethasone effect. *Cancer Chemother Pharmacol*. 2011;67(3):597-603. doi:10.1007/s00280-010-1357-2

118. Baheti G, McGuire TR, Davda JP, et al. Clinical pharmacology of etoposide in children undergoing autologous stem cell transplantation for various solid tumours. *Xenobiotica*. 2013;43(3):276-282. doi:10.3109/00498254.2012.713530

119. Veal GJ, Errington J, Sastry J, et al. Adaptive dosing of anticancer drugs in neonates: Facilitating evidence-based dosing regimens. *Cancer Chemother Pharmacol*. 2016;77(4):685-692. doi:10.1007/s00280-016-2975-0

120. Ivaturi V, Dvorak CC, Chan D, et al. Pharmacokinetics and Model-Based Dosing to Optimize Fludarabine Therapy in Pediatric Hematopoietic Cell Transplant Recipients. *Biol Blood Marrow Transpl*. 2017;23(10):1701-1713. doi:10.1016/j.bbmt.2017.06.021

121. Chung H, Hong KT, Lee JW, et al. Pharmacokinetics of fludarabine and its association with clinical outcomes in paediatric haematopoietic stem cell transplantation patients. *Bone Marrow Transpl*. 2019;54(2):284-292. doi:10.1038/s41409-018-0260-z

122. Langenhorst JB, Dorlo TPC, van Maarseveen EM, et al. Population Pharmacokinetics of Fludarabine in Children and Adults during Conditioning Prior to Allogeneic Hematopoietic Cell Transplantation. *Clin Pharmacokinet*. 2019;58(5):627-637. doi:10.1007/s40262-018-0715-9

123. Buckwalter M, Dowell JA, Korth-Bradley J, Gorovits B, Mayer PR. Pharmacokinetics of gemtuzumab ozogamicin as a single-agent treatment of pediatric patients with refractory or relapsed acute myeloid leukemia. *J Clin Pharmacol*. 2004;44(8):873-880. doi:10.1177/0091270004267595

124. Masters JC, Barry E, Knight B. Population Pharmacokinetics of Gemtuzumab Ozogamicin in Pediatric Patients with Relapsed or Refractory Acute Myeloid Leukemia. *Clin Pharmacokinet*. 2019;58(2):271-282. doi:10.1007/s40262-018-0694-x

125. Tan CT, Hancock C, Steinherz P, et al. Phase I and clinical pharmacological study of 4-demethoxydaunorubicin (idarubicin) in children with advanced cancer. *Cancer Res*. 1987;47(11):2990-2995. http://www.ncbi.nlm.nih.gov/pubmed/3471321

126. Reid JM, Pendergrass TW, Krailo MD, Hammond GD, Ames MM. Plasma pharmacokinetics and cerebrospinal fluid concentrations of idarubicin and idarubicinol in pediatric leukemia patients: a Childrens Cancer Study Group report. *Cancer Res*. 1990;50(20):6525-6528. http://www.ncbi.nlm.nih.gov/pubmed/2208112

127. Dreyer ZAE, Kadota RP, Stewart CF, et al. Phase 2 study of idarubicin in pediatric brain tumors: Pediatric Oncology Group study POG 9237. *Neuro Oncol*. 2003;5(4):261-267. doi:10.1215/S115285170200056X

128. Boddy A V, Yule SM, Wyllie R, Price L, Pearson AD, Idle JR. Pharmacokinetics and metabolism of ifosfamide administered as a continuous infusion in children. *Cancer Res*. 1993;53(16):3758-3764. http://www.ncbi.nlm.nih.gov/pubmed/8339288

129. Prasad VK, Corlett SA, Abaasi K, Heney D, Lewis I, Chrystyn H. Ifosfamide enantiomers: pharmacokinetics in children. *Cancer Chemother Pharmacol*. 1994;34(5):447-449. doi:10.1007/BF00685573

130. Boddy A V., Yule SM, Wyllie R, Price L, Pearson ADJ, Idle JR. Intrasubject variation in children of ifosfamide pharmacokinetics and metabolism during repeated administration. *Cancer Chemother Pharmacol*. 1996;38(2):147-154. doi:10.1007/s002800050463

131. Kerbusch T, de Kraker J, Mathot RAA, Beijnen JH. Population Pharmacokinetics of Ifosfamide and its Dechloroethylated and Hydroxylated Metabolites in Children with Malignant Disease. *Clin Pharmacokinet*. 2001;40(8):615-625. doi:10.2165/00003088-200140080-00005

132. Willits I, Price L, Parry A, et al. Pharmacokinetics and metabolism of ifosfamide in relation to DNA damage assessed by the COMET assay in children with cancer. *Br J Cancer*. 2005;92(9):1626-1635. doi:10.1038/sj.bjc.6602554

133. Crews KR, Stewart CF, Jones-Wallace D, et al. Altered irinotecan pharmacokinetics in pediatric high-grade glioma patients receiving enzyme-inducing anticonvulsant therapy. *Clin Cancer Res*. 2002;8(7):2202-2209. https://www.ncbi.nlm.nih.gov/pubmed/12114421

134. Gajjar A, Chintagumpala MM, Bowers DC, Jones-Wallace D, Stewart CF, Crews KR. Effect of intrapatient dosage escalation of irinotecan on its pharmacokinetics in pediatric patients who have high-grade gliomas and receive enzyme-inducing anticonvulsant therapy. *Cancer*. 2003;97(9 Suppl):2374-2380. doi:10.1002/cncr.11308

135. Vassal G, Doz F, Frappaz D, et al. A phase I study of irinotecan as a 3-week schedule in children with refractory or recurrent solid tumors. *J Clin Oncol*. 2003;21(20):3844-3852. doi:10.1200/JCO.2003.08.175

136. Wagner LM, Crews KR, Iacono LC, et al. Phase I trial of temozolomide and protracted irinotecan in pediatric patients with refractory solid tumors. *Clin Cancer Res*. 2004;10(3):840-848. doi:10.1158/1078-0432.ccr-03-0175

137. Bomgaars L, Kerr J, Berg S, Kuttesch J, Klenke R, Blaney SM. A phase I study of irinotecan administered on a weekly schedule in pediatric patients. *Pediatr Blood Cancer*. 2006;46(1):50-55. doi:10.1002/pbc.20355

138. Furman WL, Crews KR, Billups C, et al. Cefixime allows greater dose escalation of oral irinotecan: a phase I study in pediatric patients with refractory solid tumors. *J Clin Oncol*. 2006;24(4):563-570. doi:10.1200/JCO.2005.03.2847

139. Rodriguez-Galindo C, Crews KR, Stewart CF, et al. Phase I study of the combination of topotecan and irinotecan in children with refractory solid tumors. *Cancer Chemother Pharmacol*. 2006;57(1):15-24. doi:10.1007/s00280-005-0030-7

140. Bomgaars LR, Bernstein M, Krailo M, et al. Phase II trial of irinotecan in children with refractory solid tumors: a Children’s Oncology Group Study. *J Clin Oncol*. 2007;25(29):4622-4627. doi:10.1200/JCO.2007.11.6103

141. Stewart CF, Panetta JC, O’Shaughnessy MA, et al. UGT1A1 promoter genotype correlates with SN-38 pharmacokinetics, but not severe toxicity in patients receiving low-dose irinotecan. *J Clin Oncol*. 2007;25(18):2594-2600. doi:10.1200/JCO.2006.10.2301

142. Thompson PA, Gupta M, Rosner GL, et al. Pharmacokinetics of irinotecan and its metabolites in pediatric cancer patients: a report from the children’s oncology group. *Cancer Chemother Pharmacol*. 2008;62(6):1027-1037. doi:10.1007/s00280-008-0692-z

143. Furman WL, Navid F, Daw NC, et al. Tyrosine kinase inhibitor enhances the bioavailability of oral irinotecan in pediatric patients with refractory solid tumors. *J Clin Oncol*. 2009;27(27):4599-4604. doi:10.1200/JCO.2008.19.6642

144. Jannier S, Kemmel V, Sebastia Sancho C, et al. SFCE-RAPIRI Phase I Study of Rapamycin Plus Irinotecan: A New Way to Target Intra-Tumor Hypoxia in Pediatric Refractory Cancers. *Cancers (Basel)*. 2020;12(10). doi:10.3390/cancers12103051

145. Villablanca JG, Khan AA, Avramis VI, et al. Phase I trial of 13-cis-retinoic acid in children with neuroblastoma following bone marrow transplantation. *J Clin Oncol*. 1995;13(4):894-901. doi:10.1200/JCO.1995.13.4.894

146. Khan AA, Villablanca JG, Reynolds CP, Avramis VI. Pharmacokinetic studies of 13-cis-retinoic acid in pediatric patients with neuroblastoma following bone marrow transplantation. *Cancer Chemother Pharmacol*. 1996;39(1-2):34-41. doi:10.1007/s002800050535

147. Veal GJ, Cole M, Errington J, et al. Pharmacokinetics and metabolism of 13-cis-retinoic acid (isotretinoin) in children with high-risk neuroblastoma - a study of the United Kingdom Children’s Cancer Study Group. *Br J Cancer*. 2007;96(3):424-431. doi:10.1038/sj.bjc.6603554

148. Veal GJ, Errington J, Rowbotham SE, et al. Adaptive dosing approaches to the individualization of 13-cis-retinoic acid (isotretinoin) treatment for children with high-risk neuroblastoma. *Clin Cancer Res*. 2013;19(2):469-479. doi:10.1158/1078-0432.CCR-12-2225

149. Gota V, Chinnaswamy G, Vora T, et al. Pharmacokinetics and pharmacogenetics of 13-cis retinoic acid in Indian high-risk neuroblastoma patients. *Cancer Chemother Pharmacol*. 2016;78(4):763-768. doi:10.1007/s00280-016-3126-3

150. Veal GJ, Tweddle DA, Visser J, et al. Pharmacokinetics and Safety of a Novel Oral Liquid Formulation of 13-cis Retinoic Acid in Children with Neuroblastoma: A Randomized Crossover Clinical Trial. *Cancers (Basel)*. 2021;13(8). doi:10.3390/cancers13081868

151. Taha IA, Ahmad RA, Rogers DW, Pritchard J, Rogers HJ. Pharmacokinetics of melphalan in children following high-dose intravenous injection. *Cancer Chemother Pharmacol*. 1983;10(3):212-216. doi:10.1007/BF00255766

152. Ninane J, Baurain R, de Selys A, Trouet A, Cornu G. High dose melphalan in children with advanced malignant disease. A pharmacokinetic study. *Cancer Chemother Pharmacol*. 1985;15(3):263-267. doi:10.1007/BF00263898

153. Ardiet C, Tranchand B, Biron P, Rebattu P, Philip T. Pharmacokinetics of high-dose intravenous melphalan in children and adults with forced diuresis. Report in 26 cases. *Cancer Chemother Pharmacol*. 1986;16(3):300-305. doi:10.1007/BF00293997

154. Gouyette A, Hartmann O, Pico JL. Pharmacokinetics of high-dose melphalan in children and adults. *Cancer Chemother Pharmacol*. 1986;16(2):184-189. doi:10.1007/BF00256174

155. Horowitz ME, Etcubanas E, Christensen ML, et al. Phase II testing of melphalan in children with newly diagnosed rhabdomyosarcoma: a model for anticancer drug development. *J Clin Oncol*. 1988;6(2):308-314. doi:10.1200/JCO.1988.6.2.308

156. Vassal G, Tranchand B, Valteau-Couanet D, et al. Pharmacodynamics of tandem high-dose melphalan with peripheral blood stem cell transplantation in children with neuroblastoma and medulloblastoma. *Bone Marrow Transpl*. 2001;27(5):471-477. doi:10.1038/sj.bmt.1702806

157. Nath CE, Shaw PJ, Montgomery K, Earl JW. Melphalan pharmacokinetics in children with malignant disease: influence of body weight, renal function, carboplatin therapy and total body irradiation. *Br J Clin Pharmacol*. 2005;59(3):314-324. doi:10.1111/j.1365-2125.2004.02319.x

158. Nath CE, Shaw PJ, Montgomery K, Earl JW. Population pharmacokinetics of melphalan in paediatric blood or marrow transplant recipients. *Br J Clin Pharmacol*. 2007;64(2):151-164. doi:10.1111/j.1365-2125.2007.02862.x

159. Schaiquevich P, Buitrago E, Taich P, et al. Pharmacokinetic analysis of melphalan after superselective ophthalmic artery infusion in preclinical models and retinoblastoma patients. *Invest Ophthalmol Vis Sci*. 2012;53(7):4205-4212. doi:10.1167/iovs.12-9501

160. Taich P, Ceciliano A, Buitrago E, et al. Clinical pharmacokinetics of intra-arterial melphalan and topotecan combination in patients with retinoblastoma. *Ophthalmology*. 2014;121(4):889-897. doi:10.1016/j.ophtha.2013.10.045

161. Mizuno K, Dong M, Fukuda T, et al. Population Pharmacokinetics and Optimal Sampling Strategy for Model-Based Precision Dosing of Melphalan in Patients Undergoing Hematopoietic Stem Cell Transplantation. *Clin Pharmacokinet*. 2018;57(5):625-636. doi:10.1007/s40262-017-0581-x

162. Zhao J, Sharat C, Mehta PA, Mizuno K, Vinks AA, Setchell KDR. Paperspray Ionization Mass Spectrometry as a Tool for Predicting Real-Time Optimized Dosing of the Chemotherapeutic Drug Melphalan. *J Appl Lab Med*. 2021;6(3):625-636. doi:10.1093/jalm/jfaa237

163. Lennard L, Keen D, Lilleyman JS. Oral 6-mercaptopurine in childhood leukemia: Parent drug pharmacokinetics and active metabolite concentrations. *Clin Pharmacol Ther*. 1986;40(3):287-292. doi:10.1038/clpt.1986.178

164. Sulh H, Koren G, Whalen C, Soldin S, Zipursky A, Greenberg M. Pharmacokinetic determinants of 6-mercaptopurine myelotoxicity and therapeutic failure in children with acute lymphoblastic leukemia. *Clin Pharmacol Ther*. 1986;40(6):604-609. doi:10.1038/clpt.1986.233

165. Lennard L, Lilleyman JS. Variable mercaptopurine metabolism and treatment outcome in childhood lymphoblastic leukemia. *J Clin Oncol*. 1989;7(12):1816-1823. doi:10.1200/JCO.1989.7.12.1816

166. Koren G, Ferrazini G, Sulh H, et al. Systemic Exposure to Mercaptopurine as a Prognostic Factor in Acute Lymphocytic Leukemia in Children. *N Engl J Med*. 1990;323(1):17-21. doi:10.1056/NEJM199007053230104

167. Lennard L, Lilleyman JS, Van Loon J, Weinshilboum RM. Genetic variation in response to 6-mercaptopurine for childhood acute lymphoblastic leukaemia. *Lancet*. 1990;336(8709):225-229. doi:10.1016/0140-6736(90)91745-V

168. Kato Y, Matsushita T, Chiba K, Hijiya N, Yokoyama T, Ishizaki T. Dose-dependent kinetics of orally administered 6-mercaptopurine in children with leukemia. *J Pediatr*. 1991;119(2):311-316. doi:10.1016/S0022-3476(05)80751-6

169. Zuccaro P, Guandalini S, Pacifici R, et al. Fat Body Mass and Pharmacokinetics of Oral 6-Mercaptopurine in Children with Acute Lymphoblastic Leukemia. *Ther Drug Monit*. 1991;13(1):37-41. doi:10.1097/00007691-199101000-00004

170. Welch J, Lennard L, Morton GCA, Lilleyman JS. Pharmacokinetics of mercaptopurine: Plasma drug and red cell metabolite concentrations after an oral dose. *Ther Drug Monit*. 1997;19(4):382-385. doi:10.1097/00007691-199708000-00003

171. Balis FM, Holcenberg JS, Poplack DG, et al. Pharmacokinetics and pharmacodynamics of oral methotrexate and mercaptopurine in children with lower risk acute lymphoblastic leukemia: A joint Children’s Cancer Group and Pediatric Oncology Branch study. *Blood*. 1998;92(10):3569-3577. doi:10.1182/blood.v92.10.3569

172. Erb N, Harms DO, Janka-Schaub G. Pharmacokinetics and metabolism of thiopurines in children with acute lymphoblastic leukemia receiving 6-thioguanine versus 6-mercaptopurine. *Cancer Chemother Pharmacol*. 1998;42(4):266-272. doi:10.1007/s002800050816

173. Mawatari H, Unei K, Nishimura SI, Sakura N, Ueda K. Comparative pharmacokinetics of oral 6-mercaptopurine and intravenous 6-mercaptopurine riboside in children. *Pediatr Int*. 2001;43(6):673-677. doi:10.1046/j.1442-200X.2001.01475.x

174. Bell BA, Brockway GN, Shuster JJ, et al. A comparison of red blood cell thiopurine metabolites in children with acute lymphoblastic leukemia who received oral mercaptopurine twice daily or once daily: A Pediatric Oncology Group Study (Now the Children’s Oncology Group). *Pediatr Blood Cancer*. 2004;43(2):105-109. doi:10.1002/pbc.20089

175. Hawwa AF, Collier PS, Millership JS, et al. Population pharmacokinetic and pharmacogenetic analysis of 6-mercaptopurine in paediatric patients with acute lymphoblastic leukaemia. *Br J Clin Pharmacol*. 2008;66(6):826-837. doi:10.1111/j.1365-2125.2008.03281.x

176. Hanff LM, Mathot RAA, Smeets O, et al. A novel 6-mercaptopurine oral liquid formulation for pediatric acute lymphoblastic leukemia patients - Results of a randomized clinical trial. *Int J Clin Pharmacol Ther*. 2014;52(8):653-662. doi:10.5414/CP202054

177. Larsen RH, Hjalgrim LL, Grell K, et al. Pharmacokinetics of tablet and liquid formulations of oral 6-mercaptopurine in children with acute lymphoblastic leukemia. *Cancer Chemother Pharmacol*. 2020;86(1):25-32. doi:10.1007/s00280-020-04097-x

178. Pinkerton CR, Welshman SG, Kelly JG, Shanks RG, Bridges JM. Pharmacokinetics of low-dose methotrexate in children receiving maintenance therapy for acute lymphoblastic leukaemia. *Cancer Chemother Pharmacol*. 1982;10(1):36-39. doi:10.1007/BF00257235

179. Sonneveld P, Schultz FW, Nooter K, Hählen K. Pharmacokinetics of methotrexate and 7-hydroxy-methotrexate in plasma and bone marrow of children receiving low-dose oral methotrexate. *Cancer Chemother Pharmacol*. 1986;18(2):111-116. doi:10.1007/BF00262278

180. Pearson ADJ, Mills S, Amineddine HA, Long DR, Craft AW, Chessells JM. Pharmacokinetics of oral and intramuscular methotrexate in children with acute lymphoblastic leukaemia. *Cancer Chemother Pharmacol*. 1987;20(3):243-247. doi:10.1007/BF00570494

181. Balis FM, Mirro J, Reaman GH, et al. Pharmacokinetics of subcutaneous methotrexate. *J Clin Oncol*. 1988;6(12):1882-1886. doi:10.1200/JCO.1988.6.12.1882

182. Koren G, Solh H, Klein J, Soldin SJ, Greenberg M. Disposition of oral methotrexate in children with acute lymphoblastic leukemia and its relation to 6‐mercaptopurine pharmacokinetics. *Med Pediatr Oncol*. 1989;17(5-6):450-454. doi:10.1002/mpo.2950170520

183. Skoglund KA, Söderhäll S, Beck O, et al. Plasma and urine levels of methotrexate and 7‐hydroxymethotrexate in children with all during maintenance therapy with weekly oral methotrexate. *Med Pediatr Oncol*. 1994;22(3):187-193. doi:10.1002/mpo.2950220307

184. Goh TS, Wong KY, Lampkin B, O’Leary J, Gnarra D. Evaluation of 24-hour infusion of high-dose methotrexate -Pharmacokinetics and toxicity. *Cancer Chemother Pharmacol*. 1979;3(3):177-180. doi:10.1007/BF00262419

185. Ettinger LJ, Chervinsky DS, Freeman AI, Creaven PJ. Pharmacokinetics of methotrexate following intravenous and intraventricular administration in acute lymphocytic leukemia and non‐Hodgkin’s lymphoma. *Cancer*. 1982;50(9):1676-1682. doi:10.1002/1097-0142(19821101)50:9<1676::AID-CNCR2820500903>3.0.CO;2-S

186. Evans WE, Stewart CF, Chen CH, et al. Methotrexate Systemic Clearance Influences Probability of Relapse in Children With Standard-Risk Acute Lymphocytic Leukaemia. *Lancet*. 1984;323(8373):359-362. doi:10.1016/S0140-6736(84)90411-2

187. Parker RI, Forman EN, Krumm KF, Abeel MJ, Martin HF. Pharmacokinetics and Toxicity of Frequent Intermediate Dose Methotrexate Infusions. *Ther Drug Monit*. 1986;8(4):393-399. doi:10.1097/00007691-198612000-00002

188. Slørdal L, Kolmannskog S, Johan Moe P, Prytz PS, Aarbakke J. High-Dose Methotrexate Therapy (6‐8 G/M 2 ) in Childhood Malignancies: Clinical Tolerability and Pharmacokinetics. *Pediatr Hematol Oncol*. 1987;4(1):33-42. doi:10.3109/08880018709141247

189. Borsi JD, Moe PJ. A comparative study on the pharmacokinetics of methotrexate in a dose range of 0.5 g to 33.6 g/m2 in children with acute lymphoblastic leukemia. *Cancer*. 1987;60(1):5-13. doi:10.1002/1097-0142(19870701)60:1<5::AID-CNCR2820600103>3.0.CO;2-D

190. Wolfrom C, Hepp R, Hartmann R, Breithaupt H, Henze G. Pharmacokinetic study of methotrexate, folinic acid and their serum metabolites in children treated with high-dose methotrexate and leucovorin rescue. *Eur J Clin Pharmacol*. 1990;39(4):377-383. doi:10.1007/BF00315414

191. Borsi JD, Sagen E, Romslo I, Moe PJ. Comparative study on the pharmacokinetics of 7‐hydroxy‐methotrexate after administration of methotrexate in the dose range of 0.5‐33.6 g/m2 to children with acute lymphoblastic leukemia. *Med Pediatr Oncol*. 1990;18(3):217-224. doi:10.1002/mpo.2950180310

192. Najjar TAO, Al Fawaz IM. Pharmacokinetics of methotrexate in children with acute lymphocytic leukemia. *Chemotherapy*. 1993;39(4):242-247. doi:10.1159/000239132

193. Murry DJ, Synold TW, Pui CH, Rodman JH. Renal function and methotrexate clearance in children with newly diagnosed leukemia. *Pharmacotherapy*. 1995;15(2):144-149. doi:10.1002/j.1875-9114.1995.tb04347.x

194. Donelli MG, Zucchetti M, Robatto A, et al. Pharmacokinetics of HD-MTX in infants, children, and adolescents with non-B acute lymphoblastic leukemia. *Med Pediatr Oncol*. 1995;24(3):154-159. doi:10.1002/mpo.2950240303

195. Seidel H, Nygaard R, Moe PJ, Jacobsen G, Lindqvist B, Slørdal L. On the prognostic value of systemic methotrexate clearance in childhood acute lymphocytic leukemia. *Leuk Res*. 1997;21(5):429-434. doi:10.1016/S0145-2126(96)00127-0

196. Rask C, Albertioni F, Bentzen SM, Schroeder H, Peterson C. Clinical and pharmacokinetic risk factors for high-dose methotrexate- induced toxicity in children with acute lymphoblastic leukemia - A logistic regression analysis. *Acta Oncol (Madr)*. 1998;37(3):277-284. doi:10.1080/028418698429586

197. Odoul F, Guellec C Le, Lamagnère J-P, et al. Prediction of methotrexate elimination after high dose infusion in children with acute lymphoblastic leukaemia using a population pharmacokinetic approach. *Fundam Clin Pharmacol*. 1999;13(5):595-604. doi:10.1111/j.1472-8206.1999.tb00366.x

198. Wall AM, Gajjar A, Link A, Mahmoud H, Pui CH, Relling M V. Individualised methotrexate dosing in children with relapsed acute lymphoblastic leukemia. *Leukemia*. 2000;14(2):221-225. doi:10.1038/sj.leu.2401673

199. Seidel H, Andersen A, Terje Kvaløy J, et al. Variability in methotrexate serum and cerebrospinal fluid pharmacokinetics in children with acute lymphocytic leukemia: relation to assay methodology and physiological variables. *Leuk Res*. 2000;24(3):193-199. doi:10.1016/S0145-2126(99)00181-2

200. Crews KR, Liu T, Rodriguez-Galindo C, et al. High-Dose Methotrexate Pharmacokinetics and Outcome of Children and Young Adults with Osteosarcoma. *Cancer*. 2004;100(8):1724-1733. doi:10.1002/cncr.20152

201. Aumente D, Buelga DS, Lukas JC, Gomez P, Torres A, García MJ. Population pharmacokinetics of high-dose methotrexate in children with acute lymphoblastic leukaemia. *Clin Pharmacokinet*. 2006;45(12):1227-1238. doi:10.2165/00003088-200645120-00007

202. Thompson P a., Murry DJ, Rosner GL, et al. Methotrexate pharmacokinetics in infants with acute lymphoblastic leukemia. *Cancer Chemother Pharmacol*. 2007;59(6):847-853. doi:10.1007/s00280-006-0388-1

203. Piard C, Bressolle F, Fakhoury M, et al. A limited sampling strategy to estimate individual pharmacokinetic parameters of methotrexate in children with acute lymphoblastic leukemia. *Cancer Chemother Pharmacol*. 2007;60(4):609-620. doi:10.1007/s00280-006-0394-3

204. Lönnerholm G, Valsecchi MG, De Lorenzo P, et al. Pharmacokinetics of high-dose methotrexate in infants treated for acute lymphoblastic leukemia. *Pediatr Blood Cancer*. 2009;52(5):596-601. doi:10.1002/pbc.21925

205. Chládková J, Hak J, Martínková J, Chládek J. High-dose methotrexate in children with acute lymphoblastic leukemia: 7-hydroxymethotrexate systemic exposure and urinary concentrations at the steady state correlate well with those of methotrexate. *Arzneimittelforschung*. 2010;60(12):769-775. doi:10.1055/s-0031-1296353

206. Martelli N, Mathieu O, Margueritte G, et al. Methotrexate pharmacokinetics in childhood acute lymphoblastic leukaemia: A prognostic value ? *J Clin Pharm Ther*. 2011;36(2):237-245. doi:10.1111/j.1365-2710.2010.01179.x

207. Jönsson P, Skärby T, Heldrup J, Schrøder H, Höglund P. High dose methotrexate treatment in children with acute lymphoblastic leukaemia may be optimised by a weight-based dose calculation. *Pediatr Blood Cancer*. 2011;57(1):41-46. doi:10.1002/pbc.22999

208. Rühs H, Becker A, Drescher A, et al. Population PK/PD Model of Homocysteine Concentrations after High-Dose Methotrexate Treatment in Patients with Acute Lymphoblastic Leukemia. *PLoS One*. 2012;7(9). doi:10.1371/journal.pone.0046015

209. Csordas K, Hegyi M, Eipel OT, Muller J, Erdelyi DJ, Kovacs GT. Comparison of pharmacokinetics and toxicity after high-dose methotrexate treatments in children with acute lymphoblastic leukemia. *Anticancer Drugs*. 2013;24(2):189-197. doi:10.1097/CAD.0b013e32835b8662

210. Wright KD, Panetta JC, Onar-Thomas A, et al. Delayed methotrexate excretion in infants and young children with primary central nervous system tumors and postoperative fluid collections. *Cancer Chemother Pharmacol*. 2015;75(1):27-35. doi:10.1007/s00280-014-2614-6

211. Lucchesi M, Guidi M, Fonte C, et al. Pharmacokinetics of high-dose methotrexate in infants aged less than 12 months treated for aggressive brain tumors. *Cancer Chemother Pharmacol*. 2016;77(4):857-864. doi:10.1007/s00280-016-3008-8

212. Beechinor RJ, Thompson PA, Hwang MF, et al. The Population Pharmacokinetics of High-Dose Methotrexate in Infants with Acute Lymphoblastic Leukemia Highlight the Need for Bedside Individualized Dose Adjustment: A Report from the Children’s Oncology Group. *Clin Pharmacokinet*. 2019;58(7):899-910. doi:10.1007/s40262-018-00734-0

213. Medellin-Garibay SE, Hernández-Villa N, Correa-González LC, et al. Population pharmacokinetics of methotrexate in Mexican pediatric patients with acute lymphoblastic leukemia. *Cancer Chemother Pharmacol*. 2020;85(1):21-31. doi:10.1007/s00280-019-03977-1

214. Hui KH, Chu HM, Fong PS, Cheng WTF, Lam TN. Population Pharmacokinetic Study and Individual Dose Adjustments of High-Dose Methotrexate in Chinese Pediatric Patients With Acute Lymphoblastic Leukemia or Osteosarcoma. *J Clin Pharmacol*. 2019;59(4):566-577. doi:10.1002/jcph.1349

215. Kawakatsu S, Nikanjam M, Lin M, et al. Population pharmacokinetic analysis of high-dose methotrexate in pediatric and adult oncology patients. *Cancer Chemother Pharmacol*. 2019;84(6):1339-1348. doi:10.1007/s00280-019-03966-4

216. Panetta JC, Roberts JK, Huang J, et al. Pharmacokinetic basis for dosing high-dose methotrexate in infants and young children with malignant brain tumours. *Br J Clin Pharmacol*. 2020;86(2):362-371. doi:10.1111/bcp.14160

217. Shi Z yuan, Liu Y ou, Gu H yan, et al. Population pharmacokinetics of high-dose methotrexate in Chinese pediatric patients with medulloblastoma. *Biopharm Drug Dispos*. 2020;41(3):101-110. doi:10.1002/bdd.2221

218. Schulte RR, Choi L, Utreja N, Van Driest SL, Stein CM, Ho RH. Effect of SLCO1B1 Polymorphisms on High-Dose Methotrexate Clearance in Children and Young Adults With Leukemia and Lymphoblastic Lymphoma. *Clin Transl Sci*. 2021;14(1):343-353. doi:10.1111/cts.12879

219. Gao X, Qian X-W, Zhu X-H, et al. Population Pharmacokinetics of High-Dose Methotrexate in Chinese Pediatric Patients With Acute Lymphoblastic Leukemia. *Front Pharmacol*. 2021;12. doi:10.3389/fphar.2021.701452

220. Müller HJ, Löning L, Horn A, et al. Pegylated asparaginase (Oncaspar(TM)) in children with ALL: Drug monitoring in reinduction according to the ALL/NHL-BFM 95 protocols. *Br J Haematol*. 2000;110(2):379-384. doi:10.1046/j.1365-2141.2000.02187.x

221. Avramis VI, Sencer S, Periclou AP, et al. A randomized comparison of native Escherichia coli asparaginase and polyethylene glycol conjugated asparaginase for treatment of children with newly diagnosed standard-risk acute lymphoblastic leukemia: A Children’s Cancer Group study. *Blood*. 2002;99(6):1986-1994. doi:10.1182/blood.V99.6.1986

222. Pinheiro JPV, Lanvers C, Würthwein G, et al. Drug monitoring of PEG-asparaginase treatment in childhood acute lymphoblastic leukemia and non-Hodgkin’s lymphoma. *Leuk Lymphoma*. 2002;43(10):1911-1920. doi:10.1080/1042819021000015853

223. Pinheiro JPV, Wenner K, Escherich G, et al. Serum asparaginase activities and asparagine concentrations in the cerebrospinal fluid after a single infusion of 2,500 IU/m2 PEG asparaginase in children with ALL treated according to protocol COALL-06-97. *Pediatr Blood Cancer*. 2006;46(1):18-25. doi:10.1002/pbc.20406

224. Appel IM, Kazemier KM, Boos J, et al. Pharmacokinetic, pharmacodynamic and intracellular effects of PEG-asparaginase in newly diagnosed childhood acute lymphoblastic leukemia: Results from a single agent window study. *Leukemia*. 2008;22(9):1665-1679. doi:10.1038/leu.2008.165

225. Hempel G, Müller HJ, Lanvers-Kaminsky C, Würthwein G, Hoppe A, Boos J. A population pharmacokinetic model for pegylated-asparaginase in children. *Br J Haematol*. 2010;148(1):119-125. doi:10.1111/j.1365-2141.2009.07923.x

226. Tram Henriksen L, Gottschalk Højfeldt S, Schmiegelow K, et al. Prolonged first-line PEG-asparaginase treatment in pediatric acute lymphoblastic leukemia in the NOPHO ALL2008 protocol—Pharmacokinetics and antibody formation. *Pediatr Blood Cancer*. 2017;64(12):1-8. doi:10.1002/pbc.26686

227. Würthwein G, Lanvers-Kaminsky C, Hempel G, et al. Population Pharmacokinetics to Model the Time-Varying Clearance of the PEGylated Asparaginase Oncaspar® in Children with Acute Lymphoblastic Leukemia. *Eur J Drug Metab Pharmacokinet*. 2017;42(6):955-963. doi:10.1007/s13318-017-0410-5

228. Albertsen BK, Harila-Saari A, Jahnukainen K, et al. Asparaginase treatment in infants with acute lymphoblastic leukemia; pharmacokinetics and asparaginase hypersensitivity in interfant-06. *Leuk Lymphoma*. 2019;60(6):1469-1475. doi:10.1080/10428194.2018.1538507

229. Schore RJ, Devidas M, Bleyer A, et al. Plasma asparaginase activity and asparagine depletion in acute lymphoblastic leukemia patients treated with pegaspargase on Children’s Oncology Group AALL07P4*. *Leuk Lymphoma*. 2019;60(7):1740-1748. doi:10.1080/10428194.2018.1542146

230. Kloos RQH, Mathôt R, Pieters R, van der Sluis IM. Individualized dosing guidelines for PEGasparaginase and factors influencing the clearance: a population pharmacokinetic model. *Haematologica*. Published online 2020:haematol.2019.242289. doi:10.3324/haematol.2019.242289

231. Choonara I, Wheeldon J, Rayner P, Blackburn M, Lewis I. Pharmacokinetics of prednisolone in children with acute lymphoblastic leukaemia. *Cancer Chemother Pharmacol*. 1989;23(6):392-394. doi:10.1007/BF00435843

232. Hill MR, Szefler SJ, Ball BD, Bartoszek M, Brenner AM. Monitoring glucocorticoid therapy: a pharmacokinetic approach. *Clin Pharmacol Ther*. 1990;48(4):390-398. doi:10.1038/clpt.1990.167

233. Petersen KB, Jusko WJ, Rasmussen M, Schmiegelow K. Population pharmacokinetics of prednisolone in children with acute lymphoblastic leukemia. *Cancer Chemother Pharmacol*. 2003;51(6):465-473. doi:10.1007/s00280-003-0602-3

234. Sassen SDT, Mathôt RAA, Pieters R, et al. Evaluation of the pharmacokinetics of prednisolone in paediatric patients with acute lymphoblastic leukaemia treated according to Dutch Childhood Oncology Group protocols and its relation to treatment response. *Br J Haematol*. Published online 2021. doi:10.1111/bjh.17572

235. Panetta JC, Kirstein MN, Gajjar A, et al. Population pharmacokinetics of temozolomide and metabolites in infants and children with primary central nervous system tumors. *Cancer Chemother Pharmacol*. 2003;52(6):435-441. doi:10.1007/s00280-003-0670-4

236. Riccardi A, Mazzarella G, Cefalo G, et al. Pharmacokinetics of temozolomide given three times a day in pediatric and adult patients. *Cancer Chemother Pharmacol*. 2003;52(6):459-464. doi:10.1007/s00280-003-0677-x

237. Broniscer A, Iacono L, Chintagumpala M, et al. Role of temozolomide after radiotherapy for newly diagnosed diffuse brainstem glioma in children: results of a multiinstitutional study (SJHG-98). *Cancer*. 2005;103(1):133-139. doi:10.1002/cncr.20741

238. Kirstein MN, Panetta JC, Gajjar A, et al. Development of a pharmacokinetic limited sampling model for temozolomide and its active metabolite MTIC. *Cancer Chemother Pharmacol*. 2005;55(5):433-438. doi:10.1007/s00280-004-0896-9

239. Horton TM, Thompson PA, Berg SL, et al. Phase I pharmacokinetic and pharmacodynamic study of temozolomide in pediatric patients with refractory or recurrent leukemia: a Children’s Oncology Group Study. *J Clin Oncol*. 2007;25(31):4922-4928. doi:10.1200/JCO.2007.12.0667

240. Broniscer A, Gururangan S, MacDonald TJ, et al. Phase I trial of single-dose temozolomide and continuous administration of o6-benzylguanine in children with brain tumors: a pediatric brain tumor consortium report. *Clin Cancer Res*. 2007;13(22 Pt 1):6712-6718. doi:10.1158/1078-0432.CCR-07-1016

241. Meany HJ, Warren KE, Fox E, Cole DE, Aikin AA, Balis FM. Pharmacokinetics of temozolomide administered in combination with O6-benzylguanine in children and adolescents with refractory solid tumors. *Cancer Chemother Pharmacol*. 2009;65(1):137-142. doi:10.1007/s00280-009-1015-8

242. Rubie H, Geoerger B, Frappaz D, et al. Phase i study of topotecan in combination with temozolomide (TOTEM) in relapsed or refractory paediatric solid tumours. *Eur J Cancer*. 2010;46(15):2763-2770. doi:10.1016/j.ejca.2010.05.004

243. Lancaster DL, Patel N, Lennard L, Lilleyman JS. 6-Thioguanine in children with acute lymphoblastic leukaemia: Influence of food on parent drug pharmacokinetics and 6-thioguanine nucleotide concentrations. *Br J Clin Pharmacol*. 2001;51(6):531-539. doi:10.1046/j.0306-5251.2001.01391.x

244. Lowe ES, Kitchen BJ, Erdmann G, et al. Plasma pharmacokinetics and cerebrospinal fluid penetration of thioguanine in children with acute lymphoblastic leukemia: A collaborative Pediatric Oncology Branch, NCI, and Children’s Cancer Group study. *Cancer Chemother Pharmacol*. 2001;47(3):199-205. doi:10.1007/s002800000229

245. Palle J, Frost B-M, Petersson C, et al. Thioguanine pharmacokinetics in induction therapy of children with acute myeloid leukemia. *Anticancer Drugs*. 2009;20(1):7-14. doi:10.1097/CAD.0b013e32831bc086

246. Blaney SM, Balis FM, Cole DE, et al. Pediatric phase I trial and pharmacokinetic study of topotecan administered as a 24-hour continuous infusion. *Cancer Res*. 1993;53(5):1032-1036. http://www.ncbi.nlm.nih.gov/pubmed/8439950

247. Pratt CB, Stewart C, Santana VM, et al. Phase I study of topotecan for pediatric patients with malignant solid tumors. *J Clin Oncol*. 1994;12(3):539-543. doi:10.1200/JCO.1994.12.3.539

248. Stewart CF, Baker SD, Heideman RL, Jones D, Crom WR, Pratt CB. Clinical pharmacodynamics of continuous infusion topotecan in children: Systemic exposure predicts hematologic toxicity. *J Clin Oncol*. 1994;12(9):1946-1954. doi:10.1200/JCO.1994.12.9.1946

249. Baker SD, Heideman RL, Crom WR, Kuttesch JF, Gajjar A, Stewart CF. Cerebrospinal fluid pharmacokinetics and penetration of continuous infusion topotecan in children with central nervous system tumors. *Cancer Chemother Pharmacol*. 1995;37(3):195-202. doi:10.1007/BF00688317

250. Furman WL, Baker SD, Pratt CB, Rivera GK, Evans WE, Stewart CF. Escalating systemic exposure of continuous infusion topotecan in children with recurrent acute leukemia. *J Clin Oncol*. 1996;14(5):1504-1511. doi:10.1200/JCO.1996.14.5.1504

251. Tubergen DG, Stewart CF, Pratt CB, et al. Phase I Trial and Pharmacokinetic (PK) and Pharmacodynamics (PD) Study of Topotecan Using a Five-Day Course in Children with Refractory Solid Tumors. *J Pediatr Hematol Oncol*. 1996;18(4):352-361. doi:10.1097/00043426-199611000-00004

252. Frangoul H, Ames MM, Mosher RB, et al. Phase I study of topotecan administered as a 21-day continous infusion in children with recurrent solid tumors: a report from the Children’s Cancer Group. *Clin Cancer Res*. 1999;5(12):3956-3962. http://www.ncbi.nlm.nih.gov/pubmed/10632325

253. Athale UH, Stewart C, Kuttesch JF, et al. Phase I study of combination topotecan and carboplatin in pediatric solid tumors. *J Clin Oncol*. 2002;20(1):88-95. doi:10.1200/JCO.20.1.88

254. Furman WL, Stewart CF, Kirstein M, et al. Protracted intermittent schedule of topotecan in children with refractory acute leukemia: A Pediatric Oncology Group study. *J Clin Oncol*. 2002;20(6):1617-1624. doi:10.1200/JCO.20.6.1617

255. Santana VM, Zamboni WC, Kirstein MN, et al. A pilot study of protracted topotecan dosing using a pharmacokinetically guided dosing approach in children with solid tumors. *Clin Cancer Res*. 2003;9(2):633-640. http://www.ncbi.nlm.nih.gov/pubmed/12576429

256. Daw NC, Santana VM, Iacono LC, et al. Phase I and pharmacokinetic study of topotecan administered orally once daily for 5 days for 2 consecutive weeks to pediatric patients with refractory solid tumors. *J Clin Oncol*. 2004;22(5):829-837. doi:10.1200/JCO.2004.07.110

257. Stewart CF, Iacono LC, Chintagumpala M, et al. Results of a phase II upfront window of pharmacokinetically guided topotecan in high-risk medulloblastoma and supratentorial primitive neuroectodermal tumor. *J Clin Oncol*. 2004;22(16):3357-3365. doi:10.1200/JCO.2004.10.103

258. Santana VM, Furman WL, Billups CA, et al. Improved response in high-risk neuroblastoma with protracted topotecan administration using a pharmacokinetically guided dosing approach. *J Clin Oncol*. 2005;23(18):4039-4047. doi:10.1200/JCO.2005.02.097

259. Freeman BB, Iacono LC, Panetta JC, Gajjar A, Stewart CF. Using plasma topotecan pharmacokinetics to estimate topotecan exposure in cerebrospinal fluid of children with medulloblastoma. *Neuro Oncol*. 2006;8(2):89-95. doi:10.1215/15228517-2005-004

260. Schaiquevich P, Panetta JC, Iacono LC, et al. Population pharmacokinetic analysis of topotecan in pediatric cancer patients. *Clin Cancer Res*. 2007;13(22):6703-6711. doi:10.1158/1078-0432.CCR-07-1376

261. Hijiya N, Stewart CF, Zhou Y, et al. Phase II study of topotecan in combination with dexamethasone, asparaginase, and vincristine in pediatric patients with acute lymphoblastic leukemia in first relapse. *Cancer*. 2008;112(9):1983-1991. doi:10.1002/cncr.23395

262. Roberts JK, Birg A V., Lin T, et al. Population pharmacokinetics of oral topotecan in infants and very young children with brain tumors demonstrates a role of ABCG2 rs4148157 on the absorption rate constant. *Drug Metab Dispos*. 2016;44(7):1116-1122. doi:10.1124/dmd.115.068676

263. Crom WR, de Graaf SS, Synold T, et al. Pharmacokinetics of vincristine in children and adolescents with acute lymphocytic leukemia. *J Pediatr*. 1994;125(4):642-649. doi:10.1016/s0022-3476(94)70027-3

264. De Graaf SSN, Bloemhof H, Vendrig DEMM, Uges DRA. Vincristine disposition in children with acute lymphoblastic leukemia. *Med Pediatr Oncol*. 1995;24(4):235-240. doi:10.1002/mpo.2950240405

265. Gidding CEM, Meeuwsen-de Boer GJ, Koopmans P, Uges DR a., Kamps W a., de Graaf SS. Vincristine pharmacokinetics after repetitive dosing in children. *Cancer Chemother Pharmacol*. 1999;44(3):203-209. doi:10.1007/s002800050968

266. Groninger E, Meeuwsen-de Boer T, Koopmans P, et al. Pharmacokinetics of Vincristine Monotherapy in Childhood Acute Lymphoblastic Leukemia. *Pediatr Res*. 2002;52(1):113-118. doi:10.1203/00006450-200207000-00021

267. Frost BM, Lönnerholm G, Koopmans P, et al. Vincristine in childhood leukaemia: no pharmacokinetic rationale for dose reduction in adolescents. *Acta Paediatr*. 2003;92(5):551-557. http://www.ncbi.nlm.nih.gov/pubmed/12839283

268. Plasschaert SLA, Groninger E, Boezen M, et al. Influence of functional polymorphisms of the MDR1 gene on vincristine pharmacokinetics in childhood acute lymphoblastic leukemia. *Clin Pharmacol Ther*. 2004;76(3):220-229. doi:10.1016/j.clpt.2004.05.007

269. Groninger E, Meeuwsen-De Boer T, Koopmans P, et al. Vincristine pharmacokinetics and response to vincristine monotherapy in an up-front window study of the Dutch Childhood Leukaemia Study Group (DCLSG). *Eur J Cancer*. 2005;41(1):98-103. doi:10.1016/j.ejca.2004.10.006

270. Lönnerholm G, Frost B-M, Abrahamsson J, et al. Vincristine pharmacokinetics is related to clinical outcome in children with standard risk acute lymphoblastic leukemia. *Br J Haematol*. 2008;142(4):616-621. doi:10.1111/j.1365-2141.2008.07235.x

271. Guilhaumou R, Simon N, Quaranta S, et al. Population pharmacokinetics and pharmacogenetics of vincristine in paediatric patients treated for solid tumour diseases. *Cancer Chemother Pharmacol*. 2011;68(5):1191-1198. doi:10.1007/s00280-010-1541-4

272. Moore AS, Norris R, Price G, et al. Vincristine pharmacodynamics and pharmacogenetics in children with cancer: A limited-sampling, population modelling approach. *J Paediatr Child Health*. 2011;47(12):875-882. doi:10.1111/j.1440-1754.2011.02103.x

273. Lee CM, Zane NR, Veal G, Thakker DR. Physiologically Based Pharmacokinetic Models for Adults and Children Reveal a Role of Intracellular Tubulin Binding in Vincristine Disposition. *CPT Pharmacometrics Syst Pharmacol*. 2019;8(10):759-768. doi:10.1002/psp4.12453

274. van de Velde ME, Panetta JC, Wilhelm AJ, et al. Population pharmacokinetics of vincristine related to infusion duration and peripheral neuropathy in pediatric oncology patients. *Cancers (Basel)*. 2020;12(7):1-15. doi:10.3390/cancers12071789

275. Barnett S, Hellmann F, Parke E, et al. Vincristine dosing, drug exposure and therapeutic drug monitoring in neonate and infant cancer patients. *Eur J Cancer*. Accepted.
